# Analysis of Whole-Transcriptome RNA-Seq Data Reveals the Involvement of Alternative Splicing in the Drought Response of *Glycyrrhiza uralensis*

**DOI:** 10.3389/fgene.2022.885651

**Published:** 2022-05-17

**Authors:** Guozhi Li, Dengxian Xu, Gang Huang, Quan Bi, Mao Yang, Haitao Shen, Hailiang Liu

**Affiliations:** ^1^ Key Laboratory of Xinjiang Phytomedicine Resource and Utilization of Ministry of Education, College of Life Sciences, Shihezi University, Shihezi, China; ^2^ Institute for Regenerative Medicine, Shanghai East Hospital, Tongji University School of Medicine, Shanghai, China

**Keywords:** *Glycyrrhiza uralensis*, alternative splicing, drought response, transcriptome analysis, drought regulators, splicing regulatory factor

## Abstract

Alternative splicing (AS) is a post-transcriptional regulatory mechanism that increases protein diversity. There is growing evidence that AS plays an important role in regulating plant stress responses. However, the mechanism by which AS coordinates with transcriptional regulation to regulate the drought response in *Glycyrrhiza uralensis* remains unclear. In this study, we performed a genome-wide analysis of AS events in *G. uralensis* at different time points under drought stress using a high-throughput RNA sequencing approach. We detected 2,479 and 2,764 AS events in the aerial parts (AP) and underground parts (UP), respectively, of drought-stressed *G. uralensis*. Of these, last exon AS and exon skipping were the main types of AS. Overall, 2,653 genes undergoing significant AS regulation were identified from the AP and UP of *G. uralensis* exposed to drought for 2, 6, 12, and 24 h. Gene Ontology analyses indicated that AS plays an important role in the regulation of nitrogen and protein metabolism in the drought response of *G. uralensis*. Notably, the spliceosomal pathway and basal transcription factor pathway were significantly enriched with differentially spliced genes under drought stress. Genes related to splicing regulators in the AP and UP of *G. uralensis* responded to drought stress and underwent AS under drought conditions. In summary, our data suggest that drought-responsive AS directly and indirectly regulates the drought response of *G. uralensis*. Further in-depth studies on the functions and mechanisms of AS during abiotic stresses will provide new strategies for improving plant stress resistance.

## Introduction

Alternative splicing (AS) is an important post-transcriptional regulatory process that alters the structure of pre-mRNAs and regulates gene function, structure, and cellular location by increasing transcriptome plasticity and proteome diversity. During AS, exons of pre-mRNAs are spliced into different arrangements (Blencowe, 2006). In this way, AS can regulate gene transcript levels and alter the structure of transcripts and proteins (Kalyna, et al., 2012). It occurs in many tissues and at different developmental stages in eukaryotes, and is essential for coordinating plant growth and development (Dugas, et al., 2011; Gracheva, et al., 2011; Kriechbaumer, et al., 2012; Pikaard and Mittelsten, 2014). For example, AS results in different splicing variants of *ABI3* (Sugliani, et al., 2010), thereby affecting the abscisic acid signaling pathway. In wheat, different variants of *TaSSIIa-D* produced by AS determine the straight-chain starch content in the seeds (Zhou et al., 2018). The AS events associated with different developmental stages greatly affect the yield of rice (*Oryza sativa*) (Xing and Zhang., 2010). Many genes have increased the complexity of AS during domestication in maize (*Zea mays*) (Huang et al., 2015). Alternate proteins generated by AS events may compete with interacting proteins, which results in different responses to complex environmental conditions (Posé et al., 2013). Thus, AS is thought to have an important role in developmental plasticity and in the stress response in plants.

The development of high-throughput sequencing platforms has allowed for genome-wide investigations of AS events. Such studies have revealed the effect of these events on gene function, and shown that many gene transcripts undergo AS under abiotic stress conditions in plants (Feng et al., 2015; Thatcher et al., 2016; Zhu et al., 2017; Zhu et al., 2018). In *Sorghum bicolor*, for example, 2,137 AS events were detected, with intron retention (RI) being the main type of AS event (Panahi et al., 2014). In maize, 1,060 and 932 AS events were detected in the leaves and ears, respectively, during drought stress, and subsequent analyses showed that drought stress induced many developmental splicing changes in a tissue-dependent pattern (Thatcher et al., 2016). In wheat, AS regulation was found to coordinate with transcriptional regulation in response to heat, drought, and combined heat and drought (Liu et al., 2018). There is an increasing body of evidence showing that AS has broad biological significance in plants, and it can confer stress resistance (Lareau et al., 2007). For example, in *Arabidopsis* and rice, conserved AS of *HSFA2* introduces a premature translation termination codon (PTC) in the splice heterodimer *HSFA2-II,* which results in the production of truncated proteins without transcriptional activation activity under normal conditions (Cheng et al., 2015). In rice, the conserved AS of the gene encoding dehydration response element binding protein 2B (*DREB2B*) introduces a PTC into the splice isoform *OsDREB2B1* under non-stress conditions, which results in the production of a non-functional isoform. However, the functional isoform *OsDREB2B2* is significantly induced under high temperature or drought stress to enhance stress resistance (Matsukura et al., 2010). Wheat *WDREB2* is homologous to *OsDREB2B* and shows a similar AS pattern under drought conditions (Egawa et al., 2006; Terashima and Takumi, 2009). This suggests that some patterns of stress-induced AS regulation are conserved among different plant species. Therefore, an in-depth analysis of the mechanisms of AS at the post-transcriptional level is needed to understand the complex regulation of plant responses to environmental changes (Filichkin et al., 2010; Syed et al., 2012; Reddy et al., 2013). Recent research has offered a new perspective of AS as an important regulator in plants. For example, the splicing factor 3b is involved in pre-mRNA splicing related to root hair development in *Arabidopsis* in response to light signals (Ishizawa et al., 2019). Different AS messages may respond differently to stress. For example, in the GATA gene family, whose members are involved in nitrate assimilation, *OsGATA23a* shows increased transcript levels under salt and drought stress, whereas its alternatively spliced variant, *OsGATA23b*, does not respond to drought and salt stress (Gupta et al., 2017).

The legume *Glycyrrhiza uralensis* (licorice) is an important medicinal plant. It is commonly used in Chinese medicine, and its roots and rhizomes are used worldwide as a herbal medicine and natural sweetener. Its pharmacological activities include anti-inflammatory (Bai et al., 2020) and anti-cancer activities (Aipire et al., 2017). It also enhances immunomodulation, and is of high value for drug development (Hosseinzadeh and Nassiri-Asl, 2015). Although recent studies have shown that AS can regulate aspects of the plant response to drought stress (Liu et al., 2018; Song et al., 2020), there are no reports on the AS response to drought stress in upland licorice. Studies on the regulation of AS and the expression patterns of different AS isoforms under stress conditions will shed light on the mechanisms of the abiotic stress response of *G. uralensis,* and will provide clues about how AS affects the development of *G. uralensis* under abiotic stress conditions. In this study, the aboveground parts (AP) and underground parts (UP) of *G. uralensis* at 0, 2, 6, 12, and 24 h of drought stress were used as materials for RNA sequencing (RNA-seq) experiments. These analyses allowed us to identify alternatively spliced isoforms and quantify AS events at different times during the response to drought stress. We detected all five types of AS in the AP and UP of drought-stressed *G. uralensis*. Differentially spliced genes (DSGs) were identified and subjected to Gene Ontology (GO) term and Kyoto Encyclopedia of Genes and Genomes (KEGG) pathway analyses. We found that exon skipping (SE) was the main type of AS event, and that some novel AS events also played important roles in the drought response of *G. uralensis* under these conditions. These findings provide insight into the patterns of post-transcriptional AS regulation at different time points during the drought response of *G. uralensis*.

## Materials and Methods

### Plant Material and Simulated Drought Treatments

Wild *G. uralensis* (collected from the Hoboksar County Wetland Reserve in the Tacheng region of Xinjiang) was used as the experimental material. Whole seeds were treated with 98% concentrated sulfuric acid for 1 h to break seed dormancy, followed by eight rinses with distilled water. Treated seeds were germinated on vermiculite in a climate-controlled chamber (200 μmol m^−2^s^−1^ light intensity, 16 h light/8 h dark photoperiod, 50%–55% relative humidity, 28°C/25°C day/night). The plants were supplied with Murashige & Skoog (MS) nutrient solution and grown under well-watered conditions until they reached 20 cm in height (3 weeks). The *G. uralensis* seedlings were grown hydroponically for 1 week and then subjected to a simulated drought treatment (MS nutrient solution containing polyethylene glycol 6,000 at 100 g L^−1^) for 0, 2, 6, 12, and 24 h. The 0 h sample served as the control group. Samples of AP and UP were collected at 2, 6, 12, and 24 h of drought stress. The UP of the treated plants were the parts below the tiller node, and the AP were the parts above the tiller node. The UP and AP were sampled separately and preserved in liquid nitrogen until subsequent transcriptome sequencing.

### Datasets and Processing Methods

The RNA-seq dataset was analyzed to detect AS using the Repeat Multivariate Analysis of Transcript Splicing (rMATs) procedure. The transcriptomic dataset was generated from 30 libraries [(two tissues: AP and UP) × five time points during drought treatment × three replicates].

### Reference Genome-Based Assembly of Transcriptome Structures

The quality of subsequent analyses can be seriously affected by raw sequencing data containing splice sequences, low-quality reads, sequences with high N rates (where N is an uncertain base), and sequences that are too short. Therefore, to ensure the accuracy of the subsequent rMATs analyses in this study, the raw RNA-seq sequencing data were subjected to a quality control procedure to obtain high-quality data (clean data). Poor-quality bases were filtered using SeqPrep (https://github.com/jstjohn/SeqPrep) and Sickle (https://github.com/najoshi/sickle). To identify novel splice sites mapping directly to known transcripts and to generate accurate comparisons, we used TopHat2 (Kim et al., 2013) (http://ccb.jhu.edu/software/tophat/index.shtml) rather than TopHat with the licorice reference genome (*G. uralensis* Fisch*.*) for comparison. TopHat2 is a rapid alignment software developed for RNA sequencing data that first uses Bowtie2 to align RNA sequencing data to the genome and then uses the results of these alignments to detect AS events. The software String Tie (http://ccb.jhu.edu/software/stringtie/) applies a network flow algorithm and optional *de novo* assembly to assemble complex data sets into transcripts. These transcripts are then compared with known transcripts, and the novel transcripts are given MSTRG plus numbers.

### Detection and Identification of AS Events in Response to Drought Stress

The software rMATs (version 4.0.1) (http://rnaseq-mats.sourceforge.net/index.html) was used to identify AS events and to analyze differential AS events between samples. A just-only counting method was used. We identified AS events with a *p*-value false discovery rate (FDR) of <0.01 against AS events with a significant drought response. The AS events were classified as follows: SE (exon skipping), A3SS (exon AS), first exon AS (A5SS), intron retention (RI), and exon selective jumping (MXE).

### Differentially Expressed Genes Analysis

Using DESeq2 software (Love et al., 2014), RNA-seq data were analyzed in pairwise comparisons: i.e., the control group (0 h of drought treatment in AP or UP) vs. each treatment group (2, 6, 12, or 24 h of drought treatment in AP or UP) for differential gene expression analysis. Genes/transcripts with a false discovery rate (FDR) of *p*-value < 0.01 and an absolute fold change of ≥2 were identified as DEGs/transcripts. The expression levels of genes or transcripts were expressed as transcripts per million (TPM) values.

### Gene Ontology and Pathway Enrichment Analysis

The DSGs (i.e., genes from nodal reads with an FDR adjusted *p*-value <0.01 and significantly different AS events) were mapped to GO terms in the GO database (http://www.geneontology.org/) and gene numbers were calculated for each term. GO terms significantly enriched in DSGs compared with the genomic background were defined by hypergeometric tests. The *p*-values were calculated using an FDR correction (FDR cut-off ≤0.01). Significant over-representation of DSGs in KEGG pathways compared with the whole genomic background was determined by Fisher’s exact test (*p* < 0.05).

### Analysis of Expression Patterns of Genes Encoding Drought Regulators

Total RNA isolated from AP or UP of *G. uralensis* was used as the template to synthesize cDNA for RT-PCR analysis using the Plant RNA Extraction Kit (Nanjing Novozymes, Nanjing, China) (Supplementary Table S1 for primers). Then, qRT-PCR was performed using a 10-fold dilution of cDNA and SuperReal PreMix Plus (SYBR Green) (TIANGEN, Beijing, China) in a 96-well plate using the LightCycler^®^ 480 real-time PCR system (Roche Diagnostics International, Rotkreuz, Switzerland). The Log_10_2-∆∆Ct method was used to calculate relative gene transcript levels.

## Results

### Overview of RNA-Seq Data Sequencing Quality for Identifying AS Events

**FIGURE 1:**
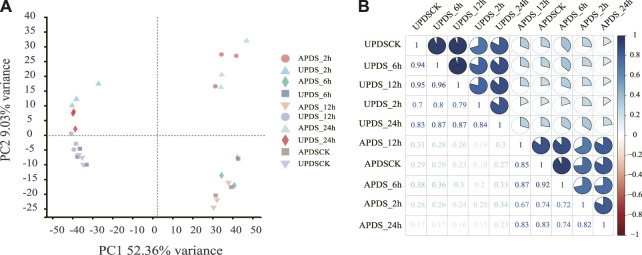
Evaluation of RNA sequence sample dataset used to identify alternative splicing (AS) events. **(A)** Principal component analysis of RNA sequence sample data. Samples from aboveground and underground parts formed different clusters at different time points (APDS_2h, APDS_6h, APDS_12h, APDS_24h, UPDS_2h, UPDS_6h, UPDS_12h and UPDS_24h). **(B)** Correlation analysis between samples. Correlation matrices were calculated by comparing the mean values of entire transcriptome in samples from aboveground and underground parts at different time points during drought treatment. Pearson’s correlation coefficients between samples were analyzed using an R script.

To study the AS and transcriptomic responses of *G. uralensis* seedlings to drought stress, we analyzed 30 RNA-seq libraries prepared from AP and UP of plants at different time points under drought stress. The libraries were generated from the following samples, in triplicate: AP of plants exposed to drought stress for 0 (APDSCK), 2 (APDS_2h), 6 (APDS_6h), 12 (APDS_12h), and 24 (APDS_24h) h; and UP of plants subjected to drought stress for 0 (UPDSCK), 2 (UPDS_2h), 6 (UPDS_6h), 12 (UPDS_12h) and 24 (UPDS_24h) h. The three biological replicates of each sample were sequenced separately. We further investigated the comprehensive profile of AS events under drought stress conditions using the RNA-seq dataset. We used TopHat2 because it produces more sensitive and accurate comparisons than TopHat, and has previously been used to analyze soybean data (Song et al., 2020). Tens of billions of clean reads were used in the downstream analysis of each sample. The Q20 (basic ratio >20), Q30 (basic ratio >30) and GC content were calculated for the clean data for each sample. The high percentage of Q30 in each sample (>96%) confirmed the high accuracy of sequencing (Supplementary Table S2). The total filtered reads were evenly distributed across the 20 chromosomes of *G. uralensis* (Supplementary Figure S1A). Analysis of the coverage of the sequencing results showed no significant bias in sequencing (Supplementary Figure S1B). Thus, the quality of the assembly and sequencing data was sufficiently high for further analyses to detect and analyze AS events. To assess the variability among samples, we performed a principal component analysis (Figure 1A). The three biological replicates formed distinct clusters, indicating that there was an acceptable amount of variation among replicates at any selected time point. In addition, the correlation coefficients (*r* ^2^) for the transcriptomes in AP and UP were 0.67–1 and 0.7–1, respectively, (Figure 1B), indicating that the expression patterns were relatively similar among the 15 AP samples and among the 15 UP samples. This further confirmed the reliability of the sequencing data.

### Identification of Differential AS Events in AP and UP of *G. uralensis* at Different Time Points During Drought Treatment

**FIGURE 2:**
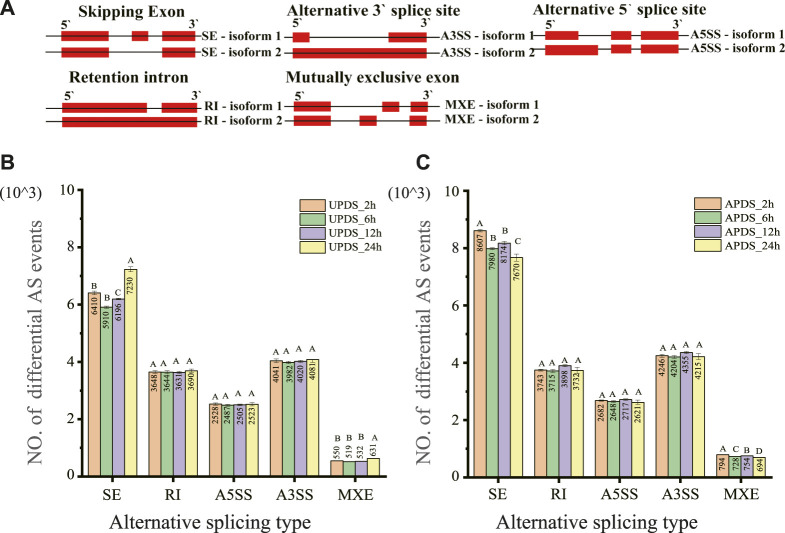
Classes and numbers of different types of AS events detected in the transcriptome of *Glycyrrhiza uralensis* at different time points during drought treatment. **(A)** Schematic diagram of the AS: SE, A3SS, A5SS, RI, and MXE. Each event produces two types of isomers: isoforms 1 and 2. **(B)** Number of five different types of AS events in AP of *G. uralensis* at different time points under drought stress. **(C)** Number of five different types of AS events in UP of *G. uralensis* at different time points under drought stress. Data are mean ± standard deviation of three biological replicates. Mean values for different time points under drought stress were tested using the Bonferroni test. Different letters indicate highly significant differences among treatments.

High-quality reads were first mapped to the *Glycyrrhiza* reference genome (species name: *Glycyrrhiza_uralensis* Reference genome version: Riken Reference genome source: http://ngs-data-archive.psc.riken.jp/Gur-genome/index.pl) (Mochida et al., 2017) and then AS events were identified and quantified by rMATS (Junction Count Only setting). All five AS types were identified in AP and UP of *G. uralensis* at different time points during the drought stress treatment (Figure 2A). Using the AP and UP datasets, we applied the Kolmogorov–Smirnov method to determine the number of each of the five AS types at each time point with a positive-terrestrial distribution during the drought treatment (Supplementary Table S3). The mean values were subjected to one-way ANOVA after correction using the Bonferroni method (Supplementary Table S4). The number of A3SS, A5SS, and RI-type AS events did not differ significantly between the AP and UP of *G. uralensis* at 2, 6, 12, and 24 h of drought stress. The number of SE-type AS events differed significantly (*p* < 0.01) between 24 h of drought stress and 2, 6, and 12 h of drought stress in both AP and UP, but not between 6 and 12 h of drought stress. The number of SE and MXE-type AS events in UP differed significantly (*p* < 0.01) among 2, 6, and 24 h of drought stress. The numbers of all five AS events were significantly higher in AP than in UP. The number of AS events tended to decrease and then increase during the drought treatment (Figures 2B,C). The proportions of the five AS events were similar in AP and UP. Of the five types of AS events, SE was the most abundant (35%–38%), followed by A3SS (22%–24%), A5SS (15%), and RI (20%–22%). The rarest event was MXE (4%–5%) (Supplementary Figure S2A). These results show that AS events commonly occurred under drought stress, and SE was the main type of differential AS event in AP and UP during the response to drought stress in *G. uralensis*.

### Identifying AS Events in Response to Drought Stress in AP and UP of *G. uralensis*

**FIGURE 3:**
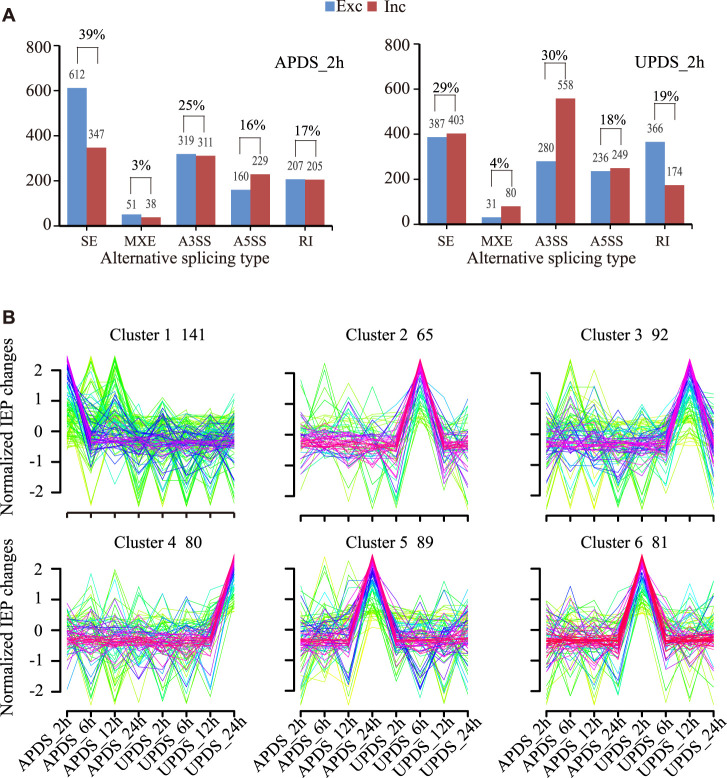
Identification and comparative analysis of stress-responsive AS events in *G. uralensis*. **(A)** Comparison of number and proportion of different AS events in different parts of *G. uralensis*. Exclusion is shown in blue, inclusion in red. *y*-axis shows number of AS events, *x*-axis shows types of AS events. **(B)** Cluster analysis of stress-responsive AS events in *G. uralensis*, as indicated by changes in isoform expression percentage (IEP) under various drought conditions. [IEP = mean PSI (ck)/(mean PSI (ck) + mean PSI (stress)]. Number of AS events in each cluster is listed. *x*-axis shows various stress conditions and *y*-axis shows IEP values. Red line shows the trend of average IEP value for all AS events in each cluster.

Previous studies have shown that drought stress-responsive AS events lead to the production of differently spliced isoforms of genes under stress in soybean (Song et al., 2020). Such events are also thought to be involved in regulating the response of *G. uralensis* to drought stress. Here, the criterion for a drought stress-responsive AS event was an FDR (adjusted *p*-value) of <0.05 based on nodal readings only. In total, 2,479 and 2,764 drought stress-responsive AS events were identified in the AP and UP of *G. uralensis*, respectively, consisting of 959, 89, 630, 389, and 412 SE, MXE, A3SS, A5SS, and RI-type AS events, respectively, in AP; and 790, 111, 838, 485, and 540 SE, MXE, A3SS, A5SS, and RI-type AS events, respectively, in UP. This result indicates that the AS response was enhanced in UP compared with AP in drought-stressed *G. uralensis* (Figure 3A). Among all the AS events, the most abundant types were SE (39% in AP, 29% in UP) and A3SS (25% in AP, 30% in UP), consistent with previous reports (Song et al., 2020). There were significantly more exclusion-type SE events than inclusion-type SE events in AP, but there was no difference in their relative frequencies in UP. In addition, there were significantly more intrinsic A3SS events than excluded A3SS events in UP, but there was no difference in their relative frequencies in AP (Figure 3A). Thus, the distribution of AS types and the proportions of different isomers differed among different parts of licorice plants.

Changes in the isoform expression percentage (IEP) have been used to group stress-responsive AS events in wheat (Liu. et al., 2018) and soybean (Song et al., 2020). The SE-type events are the main type of stress-related AS event. We performed a cluster analysis of IEP changes in SE stress-responsive AS events using the Mfuzz program (Figure 3B; Supplementary Table S5) (Kumar and Futschik et al., 2007). The criteria for the definition of IEP were “FDR (adjusted *p*-value) <0.05 based on node-only readings” and “≥30% change in isoform expression percentage [IEP = mean PSI (percent spliced in) (ck)/(mean PSI (ck) + mean PSI (stress)]” after stress treatment. Six groups were identified based on the drought response characteristics of AS. Group 1, with a total of 141 AS events, represented the change in the AS pattern in the APDS_2h treatment; Group 2 (65 AS events) represented minor changes in the AS pattern in the UPDS_6h group. Groups 3, 4, 5, and 6 represented the change in AS pattern in the UPDS_12h treatment, the UPDS_24h treatment, the APDS_24h treatment, and the UPDS_2h treatment, respectively. Thus, the changes in AS patterns in response to drought stress occurred mainly in the UP of *G. uralensis*. Our results revealed six groups of AS events in response to drought stress at different time points.

### Comparative Analysis of Differentially Expressed and Differentially Spliced Genes

**FIGURE 4:**
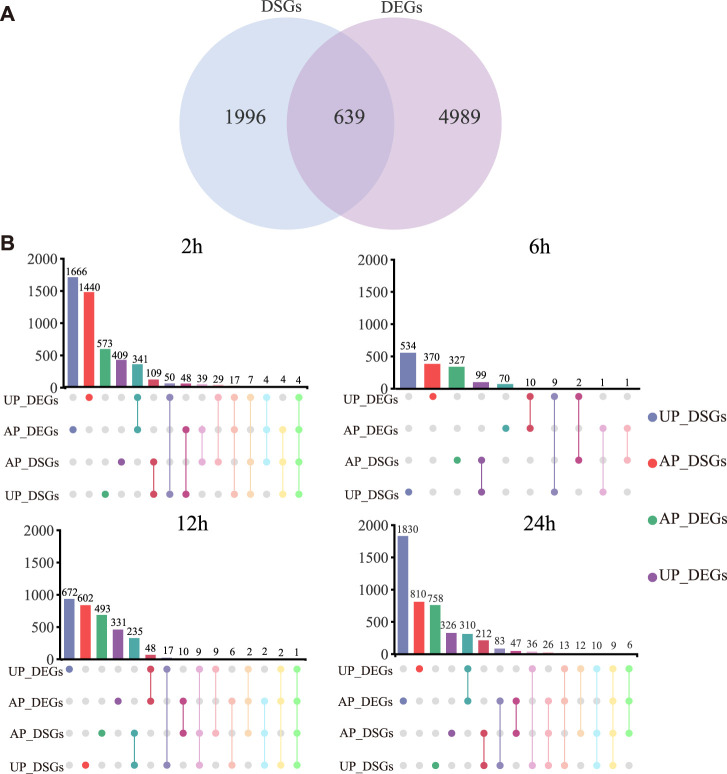
Comparative analysis of differentially spliced genes (DSGs) and differentially expressed genes (DEGs) in *G. uralensis* at different time points under drought stress. **(A)** Comparative analysis of overlapping DEGs and DSGs under all drought conditions. **(B)** Number of DSGs and DEGs in AP and UP, and those overlapping between AP and UP, at different time points during the drought treatment.

DSGs are genes that are affected by AS events, in this case, under drought stress. We detected a total of 2,653 *G. uralensis* DSGs (596, 427, 753, 644, 804, 634, 866, and 1,133 at different time points during the drought treatment) (Supplementary Table S6). In total, 639 genes exhibited distinct drought-responsive expression patterns and AS regulation patterns (Figure 4A). Second, to investigate the changes in AS after drought stress, the number of DSGs was compared with the number of DEGs in UP and AP of *G. uralensis* at different times during the drought treatment (Figure 4B; Supplementary Table S6). At 2, 6, 12, and 24 h of drought stress, 39, 1, 10, and 47 genes, respectively, were both DSGs and DEGs in AP, indicative of significant drought-responsive expression patterns and AS regulation; and 50, 9, 17, and 36 genes, respectively, were both DSGs and DEGs in UP (Figure 4B). Therefore, in AP and UP, AS regulation occurred most strongly at 2 and 24 h of drought stress, and more weakly at 6 h of drought stress. These results highlight that some stress-responsive genes are further modified by AS in response to drought stress.

To further investigate whether new AS events were identified and involved in the drought response of *G. uralensis*, we examined three AS (MXE, RI, and SE) events that were significantly responsive to drought stress (Supplementary Table S7). First, more novel AS events were identified in UP than in AP. In total, 1,312 novel SE-type AS events were identified across all drought-stressed samples, suggesting that SE-type AS is strongly involved in the drought response of *G. uralensis* (Supplementary Table S7)*.*

### Biological Functions of DSGs Regulated at AS and Transcriptional Levels

**FIGURE 5:**
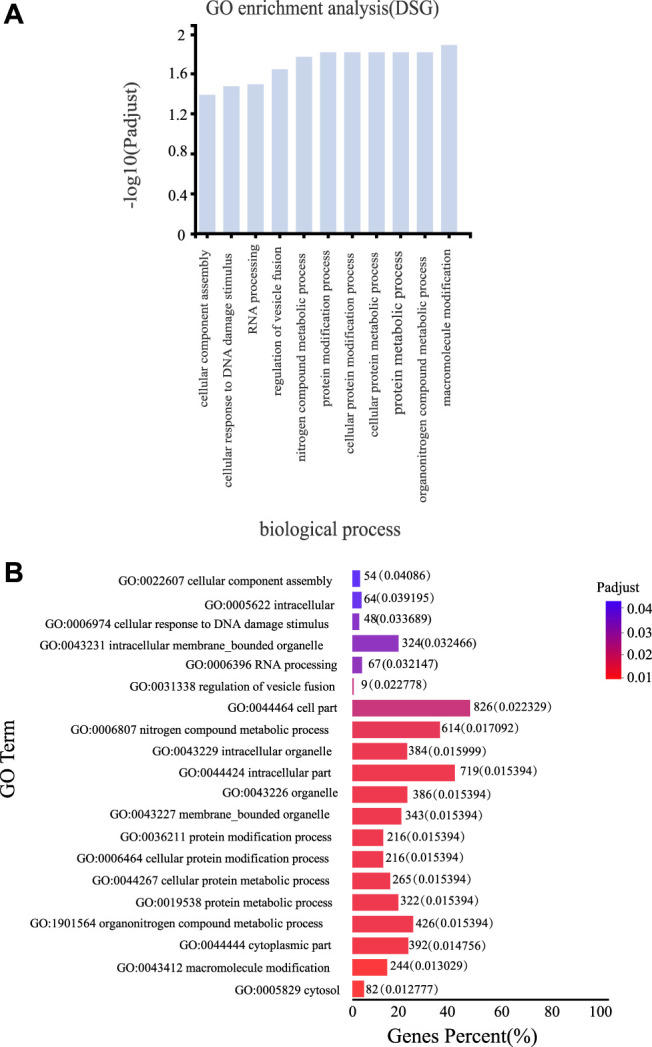
Gene Ontology (GO) analyses of DSGs. **(A)** GO terms in the biological process and cellular component categories assigned to DSGs under various drought conditions [false discovery rate (FDR) values <0.05]. **(B)** Top 20 GO terms (molecular function) enriched with DSGs under different drought conditions. Note: Histograms represent the percentage of genes (%) for GO terms.

To determine how AS regulation affects the biological functions of licorice plants in response to drought, we performed GO enrichment analysis on all the DSGs. These analyses revealed that many genes involved in metabolic and cellular biological processes were regulated by AS (Figure 5A). The following GO terms were enriched with DSGs: macromolecule modification, organonitrogen compound metabolic process, protein metabolic process, cellular protein metabolic process, cellular protein modification process, protein modification process, and nitrogen compound metabolic process. These findings suggested that AS is involved regulating protein modification and metabolism during the drought response of *G. uralensis* (Figure 5B). The significantly enriched biological process GO terms assigned to the DSGs were related to intracellular organelles and the cytoplasm and included the following: cytosol, cytoplasmic part, membrane-bounded organelle, organelle, intracellular part, intracellular organelle, cell part, regulation of vesicle fusion, intracellular membrane-bound organelle, intracellular, and cellular component assembly. These results suggested that AS is involved in regulating intracellular biological processes and biochemical activities, thereby playing an important role in the drought response of *G. uralensis* (Figure 5B). Other subcategories significantly enriched with DSGs were material metabolic processes and major metabolic processes (Supplementary Figure S3). An understanding of these enriched biological processes, metabolic pathways, and biochemical activities provides an overview of the changes that occur as a result of AS regulation during the drought response in *G. uralensis*.

### Comparative Analysis of DEGs and DSGs Encoding Splicing Regulators

**FIGURE 6:**
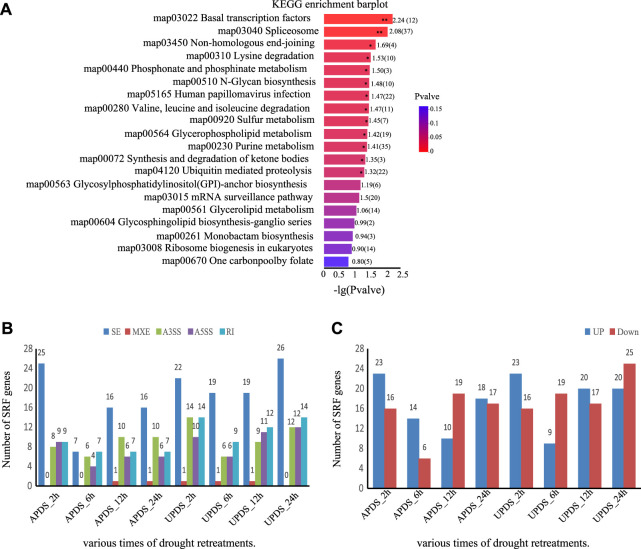
Expression and AS analysis of SPF-related genes in different drought treatments. **(A)** Top 20 pathways enriched with DSGs. −Log10 (*p*-value) expresses the ratio of the number of DSGs to the number of genes annotated in the pathway. The larger the −Log10 (*p*-value), the greater the enrichment. **, *p* < 0.01; *, *p* < 0.05. **(B)** Number of significantly differentially spliced SPF-associated genes at different time points under drought stress. **(C)** Number of significantly differentially expressed SPF-associated genes at different times points under drought stress.

Splicing regulatory factors (SPFs), such as heterogeneous ribonucleic acid proteins (hnRNPs), pre-mRNA splicing factors, and Ser/Arg-rich proteins, not only respond to drought stress, but also undergo variable splicing under various abiotic and biotic stress conditions (Palusa et al., 2007; Tanabe et al., 2007; Filichkin et al., 2010; Ding et al., 2014). We applied KEGG pathway analysis to determine which pathways were enriched with these DSGs. We found that the spliceosome pathway (*p*-value < 0.0057) and basal transcription factors pathway (*p*-value< 0.0083) were enriched with DSGs under drought stress (Figure 6A). These results suggest that genes encoding SPFs respond to drought stress in *G. uralensis* and undergo variable splicing.

In this study, 37 SPF-related genes showed significant splicing differences among different time points during drought treatment. The main AS events were SE and A3SS types (Figure 6B). There were more upregulated SPF-related genes than downregulated ones in all treatments except for APDS_12h and UPDS_6h (Figure 6C). The responses of SPF-related genes to drought treatment differed significantly among different time points. The majority of SPF-related genes were upregulated in the UPDS_6h, UPDS_12h, and UPDS_24h treatments (Supplementary Figure S4). These results suggest that genes encoding SPFs are themselves under AS regulation during the response to drought stress in the roots of licorice plants.

To further reveal how stress-responsive genes in UP of *G. uralensis* respond to drought stress, we investigated the expression patterns of four genes encoding SPFs in detail. As shown in Supplementary Figure S5, at different time points under drought stress, Glyur000404s00017726 (encoding Sm-like protein LSM2), Glyur000842s00023837 (encoding serine/arginine-rich splicing factor RS2Z33), and Glyur000784s00031233 (encoding phosphatidylinositol-glycan biosynthesis class F protein) were weakly expressed as isoform 1 (full-length protein-encoding isoform) but strongly expressed as isoform 2 (sheared protein-encoding isoform that was sheared) (Supplementary Figures S5A,C,D). In contrast, the Glyur000959s00024505 gene (Protein RDM16) was mainly expressed as isoform 1, with few transcripts of isoform 2 (Supplementary Figure S5B). Thus, different genes respond differently to drought stress and, therefore, may play different roles in regulating the drought response of *G. uralensis*.

### Identifying Potential Drought Regulators in the Response to Drought Stress in *G. uralensis*

Many genes encoding core proteins in the ABA signaling pathway, drought-associated cascade proteins, and heat-strike associated proteins showed significant changes in AS patterns (Supplementary Figure S6). For example, genes encoding the 18.1 kDa class I heat shock protein and 22 kDa heat shock protein underwent different AS events (Supplementary Figures S6A,B). Moreover, genes encoding the drought-induced probable WRKY transcription factors 19 and 20 showed distinct changes in AS patterns at different timepoints under drought stress (Supplementary Figures S6C,D). At different times of drought stress, genes encoding core proteins in the ABA signaling pathway, probable protein phosphatase 2C 76, probable protein phosphatase 2C 6, protein phosphatase 2C 16, and probable protein phosphatase 2C 26 also showed different types of AS modifications (Supplementary Figures S6E–H). Thus, many of the pathways enriched with DEGs were also enriched with DSGs.

In AP, the HSP22 gene expression levels at APDS_2h and APDS_24h were significantly higher than those at the other treatment time points (*p* < 0.01), and were significantly higher than those at UPDS_2h and UPDS_24h (*p* < 0.01). The WRKY19 gene expression levels at APDS_2h and APDS_24h were significantly higher than those at the other treatment time points (*p* < 0.01), and were significantly higher than those at UPDS_2h and UPDS_24h (*p* < 0.01). The WRKY20 gene expression levels at APDS_2h, APDS_12h, and APDS_24h were significantly lower than those following the control treatment (*p* < 0.01). The PP2C gene expression levels at APDS_2h, APDS_6h, and APDS_12h were significantly higher than those in the control group (*p* < 0.01), and were significantly higher than those at UPDS_2h and UPDS_12h (*p* < 0.01). In UP, the HSP22 gene expression level was significantly higher at UPDS_2h than at the other treatment time points (*p* < 0.01). There were no significant differences in the WRKY19 gene expression levels among the different stress treatments. The WRKY20 gene expression levels at UPDS_6h, UPDS_12h, and UPDS_24h were significantly higher than those after the control treatment (*p* < 0.05). The PP2C gene expression levels at UPDS_2h, UPDS_12h, and UPDS_24h were significantly lower than those after the control treatment (*p* < 0.01). The HSP22, WRKY19, WRKY20, and PP2C gene expression levels were significantly higher in AP than in UP (Supplementary Figure S7). Thus, the significant alternative splicing events that affected HSP22, WRKY19, WRKY20, and PP2C gene expression in UP resulted in the increased production of proteins to cope with the exposure to drought stress. Moreover, this further verifies the reliability of our results.

**FIGURE 7:**
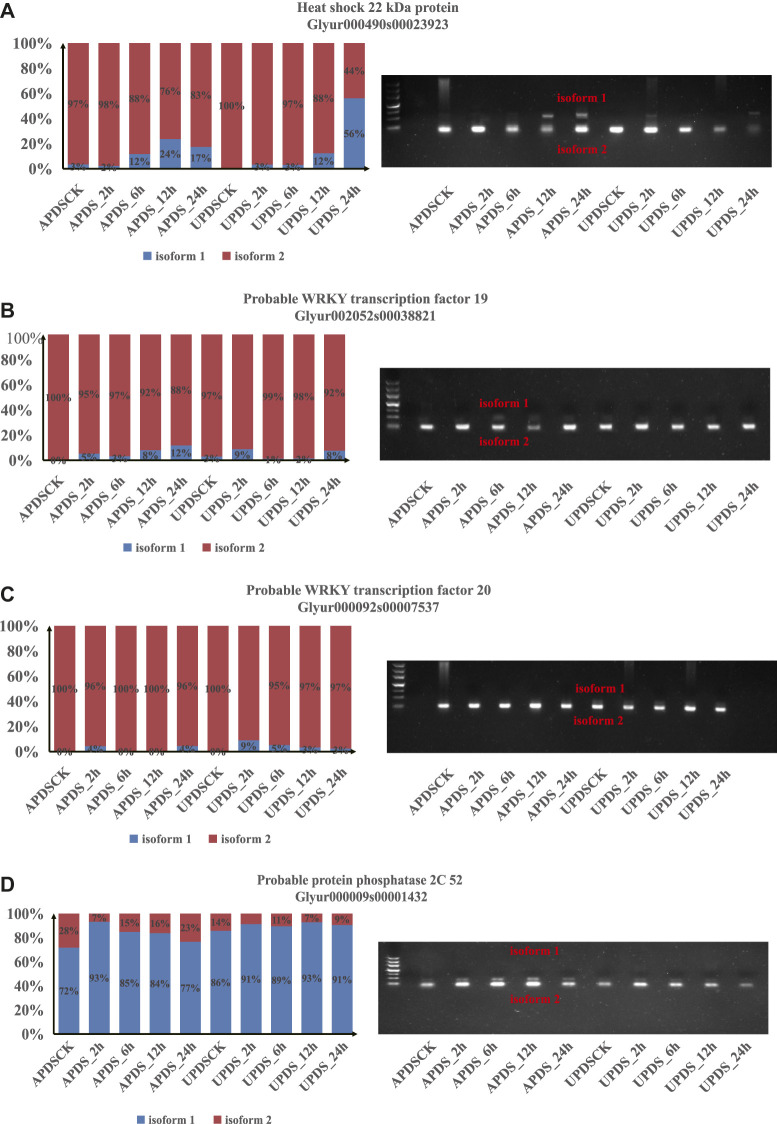
Experimental validation of stress response events in licorice AP and UP by RT-PCR. **(A)** Gene encoding 22 kDa heat shock protein; **(B)** Gene encoding probable WRKY transcription factor 19; **(C)** Gene encoding probable transcription factor 20; **(D)** Gene encoding probably protein phosphatase 2C 52. In the left panels, bars show relative expression levels of alternatively spliced isoforms 1 (blue) and 2 (red) in AS at 0, 2, 6, 12, and 24 h of drought stress as determined from RNA-seq data. Panels on the right show results of RT-PCR analyses to detect splice variants using specific primer pairs.

To confirm the patterns of AS events under drought stress identified by RNA sequencing, we further validated the predicted AS patterns by reverse transcription RT-PCR and real-time fluorescent quantitative PCR (Figure 7). Conservative primer pairs were used to amplify two splice variants (isoforms 1 and 2) in a single reaction. As expected, the four validated DSGs, including one HSP gene, two WRKY genes and one PP2C gene, showed AS patterns consistent with those detected from the RNA-Seq data, further confirming the accuracy of our bioinformatics analyses (Figure 7). For example, the RNA-Seq data indicated that isoform 2 of HSP22 (Glyur00490s00023923) was significantly induced in AP at 0, 2, and 6 h of drought stress and in UP at 0, 2, 6, and 12 h of drought stress, whereas its isoform 1 was induced in UP at 24 h of drought stress and in AP at 12 h of drought stress (Figure 7A). In the PCR analyses, we detected isoform 2 bands of HSP22 in AP at 0, 2, and 6 h of drought stress and in UP 0, 2, 6, and 12 h of drought stress, whereas isoform 1 and 2 bands were amplified from UP at 24 h of drought stress and AP at 12 h of drought stress (Figure 7A).

## Discussion

### Many AS Events Occur in *G. uralensis* at Different Time Points Under Drought Stress

Previous studies have shown that AS events in plants participate in the regulation of stress responses. However, abiotic stress-regulated AS events have not been systematically analyzed and reported at the whole-transcriptome level for licorice (Ding et al., 2014; Thatcher et al., 2016; Calixto et al., 2018; Liu et al., 2018; Li et al., 2020). In this study, using whole-transcriptome RNA-seq data, 2,479 and 2,764 AS events in response to drought stress were identified in *G. uralensis* AP and UP, respectively, at 2 h of drought stress, consisting of 959, 89, 630, 389, and 412 SE, MXE, A3SS, A5SS, and RI-type AS events, respectively, in AP; and 790, 111, 838, 485 and 540 SE, MXE, A3SS, A5SS, and RI-type AS events, respectively, in UP. There were more AS stress response events in UP than in AP, indicating that the AS response was stronger in UP than in AP under drought stress. Thus, our results not only illustrate that many diverse AS events occur in *G. uralensis* at different times under drought stress, but also support the important role of AS events in regulating the drought response of *G. uralensis*.

### A3SS and SE are the Main AS Types in *G. uralensis* in Response to Drought Stress

Although we detected all five AS types in drought-stressed licorice plants, SE and A3SS were the most abundant types (Supplementary Figure S2). These two types of AS accounted for 59%–64% of all drought stress responsive AS events (Figure 3). At 2, 6, 12, and 24 h of drought stress, there were more RI events than A5SS events (Figure 2), but approximately equal numbers of A5SS events (389–485) and RI events (412–540) (Figure 3). This result is consistent with the results of another study, where SE and A3SS were found to be the most abundant AS types in soybean in response to drought stress (Shen et al., 2014; Wang et al., 2014; Iñiguez et al., 2017). In contrast, RI was identified as the most abundant AS event at different developmental stages in soybean. In wheat, RI was found to be the dominant AS type responding to drought, high temperature, and combined heat and drought, whereas in maize, it was the main type of AS in response to nitrogen (Liu et al., 2018; Wang et al., 2020). There will be similarities in the main AS types in response to drought stress in closely related species. Differences in the dominant AS types among different developmental stages and among distantly related species may be related to the regulation of SPF-related genes (including gene expression levels and splicing events). Further work is needed to validate and explain these observed differences.

### Gene Ontology Terms and Kyoto Encyclopedia of Genes and Genomes Pathways Enriched With Differentially spliced genes

In previous studies, DEGs under drought stress were found to be associated with metabolic pathways and with the biosynthesis of secondary metabolites and phenylpropanoids (Song et al., 2016). In this study, some DSGs were associated with the spliceosome and basal transcription factor pathways (Figure 6). In the KEGG analysis in this study, 49 (4%) of the DSGs were associated with the spliceosome pathway (*p-*value < 0.0057) and the basal transcription factor pathway. Spliceosomes process pre-RNAs into mature mRNAs, so they are an essential component of the splicing process (Syed et al., 2012). In *Arabidopsis*, modifications of key spliceosomal components were reported to be consistent with transcriptional and proteomic changes that occurred under drought stress (Marondedze et al., 2020). In the present study, genes encoding several RNA-binding proteins (Glyur000002s00000283, Glyur002500s00036004, and Glyur000130s00006911) were among those regulated at the AS level, although the detailed composition of the *G. uralensis* spliceosome under drought stress has not been described. Thus, specific spliceosomal changes also appear to occur in *G. uralensis* under drought stress.

Basal transcription factors are essential for the function of RNA polymerase II. Twenty nine years ago, the transcription factor IIH (TFIIH) multiprotein complex was found to link transcription and nucleotide excision repair processes (Schaeffer et al., 1993). Although many studies have explored the function of TFIIH, the exact events that occur between transcription and DNA repair are still unknown. In addition, several studies have revealed that components of TFIIH are involved in other processes such as the cell cycle and chromosome segregation (DiGiovanna and Kraemer, 2012; Rapin, 2013). In cotton, CDK (CDKF4) has been reported to be strongly induced by drought stress; CDKs are important not only in cellular regulation but also in stress resistance. *Arabidopsis* lines overexpressing CDKF4 showed increased expression of RD29A, CBL1, and ABF4, and CDKF4 was found to have great potential in enhancing tolerance to various abiotic stresses (Magwanga et al., 2018). In our study, two genes encoding cyclin-dependent kinase D-1 (CDK7) (Glyur002778s00034252 and Glyur000074s00005568) were among those regulated by AS under drought stress. Thus, basal transcription factors may also be involved in regulating the drought response of *G. uralensis*.

Plant splicing regulators are essential for regulating the AS of drought-related genes (Palusa et al., 2007; Tanabe et al., 2007; Filichkin et al., 2010; Ding et al., 2014). The interaction between Ser/Arg-rich proteins and pre-mRNA is essential for constitutive and AS and contributes to maintaining cellular and tissue homeostasis (Reddy and Shad Ali, 2011; Ding et al., 2014; Laloum et al., 2018). Interestingly, plant SPF-related genes are themselves subject to AS in a developmental and tissue-specific manner, as well as in response to various hormonal and abiotic stresses (Zhang and Mount, 2009; Ding et al., 2014; Zhu et al., 2018). In other studies, overexpression of BrSR45a in *Arabidopsis* not only increased the abundance of drought stress-inducible genes, but also affected the splicing pattern of target genes (Muthusamy et al., 2020). The abundance of transcripts and the proportion of exon deletions in AtSR45a in *Arabidopsis* was found to be affected by heat and drought stress (Gulledge et al., 2012). Notably, some genes encoding Ser/Arg-rich proteins involved in pre-mRNA splicing were affected by AS events under drought stress in this study (Figure 6), such as Glyur000221s00012517 (a homolog of SR45a). It is possible that stress-dependent AS of SPF-related genes also plays an important role in regulating the drought response of *G. uralensis*.

The GO terms significantly enriched with DSGs included those related to nitrogen and protein metabolism (Figure 5B). In apple, genes related to nitrogen, secondary compounds, and amino acid metabolism were found to be involved in the response to drought stress (Gao et al., 2020). Thus, in addition to changes in gene expression levels, AS events may also play an important role in the drought response of licorice. The enriched pathways under drought stress may be conserved among different plant species.

### Important Regulatory Role of AS in Plant Development and in the Response to Abiotic Stresses

AS has been extensively studied in several plant species. Gene editing technology [Clusters of Regularly Interspaced Short Palindromic Repeats, (CRISPR)] has been used to identify AS events and gene functions. Splice site recognition sequences differ between plants and animals (Reddy, 2004; Moore and Proudfoot., 2009; Filichkin et al., 2010). This may be because plants must endure unfavorable environmental conditions because of their sessile growth habit. The AS regulation of genes encoding key abiotic stress regulators, such as the heat stress transcription factor (HsfA2) and dehydration response element binding protein 2B (DREB2B), are conserved across plant species (Egawa et al., 2006; Terashima and Takumi, 2009; Hu et al., 2020). Environmental stress affects these AS regulators and further regulates the expression of AS and other stress-related genes in plants, thus ensuring that plants can respond and adapt rapidly to environmental changes (Achard et al., 2006; Baena-González et al., 2007; Barta et al., 2010; Jia et al., 2012; Scharf et al., 2012). Recent transcriptomic and proteomic analyses of 6-month-old *Quercus ilex* seedlings under severe drought conditions revealed a decrease in the accumulation of metabolism-related transcripts and proteins, but an increase in the accumulation of stress-related transcripts and proteins (e.g., HSP22) (Guerrero-Sánchez et al., 2021). The overexpression of the *A. thaliana* WRKY30 (AtWRKY30) transcription factor in wheat under drought and high-temperature stress conditions revealed that the expression levels of antioxidant enzyme genes and the stress-responsive WRKY19 gene are significantly higher in transgenic wheat plants than in wild-type plants, enabling the transgenic plants to limit the damages caused by drought and high temperatures (El-Esawi et al., 2019). In an earlier investigation of drought stress, 42 RNA-seq samples from the following seven plant species were analyzed: *A. thaliana*, *Solanum lycopersicum*, *Zea mays*, *Vitis vinifera*, *Malus* × *domestica*, *Solanum tuberosum*, and *Triticum aestivum*. The WRKY20 protein was determined to play a key role in the drought response of leaves (Benny et al., 2019). In response to drought stress, SnRK2s phosphorylate several transcription factors that activate the transcription of ABA-responsive genes encoding proteins implicated in stress responses and stress tolerance. In contrast, the clade A type 2C protein phosphatases (PP2Cs) inhibit SnRK2s *via* a physical interaction, thereby negatively regulating ABA signaling (Umezawa et al., 2009). Under drought stress conditions, PP2Cs bind to ABA receptors to capture ABA while also releasing and activating SnRK2s. Thus, PP2Cs function as a switch in the central part of the ABA signaling network (Jung et al., 2020). In our study, the HSP22, WRKY19, WRKY20, and PP2C gene expression levels were significantly higher in AP than in UP (Supplementary Figure S7). Additionally, HSP22, WRKY19, WRKY20, and PP2C gene expression was modified by significant alternative splicing events in UP, which led to an increase in the production of proteins that mitigated the adverse effects of drought stress. The reliability of our analysis was further verified. With the development of high-throughput sequencing technology, large amounts of plant genome sequence and transcriptome data are available for analyses of AS events and how they differ among species, tissues, and developmental stages. Analyses of such datasets can also reveal how AS patterns change under various environmental conditions. Studies on the function and mechanisms of AS during abiotic stress could provide new strategies for improving plant stress resistance. In summary, our results highlight that a large number of AS events occur in *G. uralensis* under drought stress. Our analyses of whole-transcriptome RNA-seq data from AP and UP at 2, 6, 12, and 24 h of drought have provided a comprehensive view of AS in *G. uralensis* at different time points under drought stress. Thus, in addition to differential gene expression, drought-responsive AS events and/or their expression levels directly or indirectly regulate the drought response of *G. uralensis*. Further systematic approaches are needed to dissect and understand the response of *G. uralensis* to drought stress and the roles of specific DSGs and DEGs in the drought stress response.

## Data Availability

The datasets presented in this study can be found in online repositories. The names of the repository/repositories and accession number(s) can be found in the article/Supplementary Material.

## Supplementary Material

Supplementary Figure S1 Quality and characteristics of the RNA-seq dataset. **(A)** Distribution of all filtered reads on the first 20 large chromosomal segments in *G. uralensis*. Reads were obtained across the entire licorice genome and were distributed on different large chromosomal segments. Raw reads were filtered according to the following criteria: Mapping Qualities (MAPQ) > 20 and an insert size of 100–1000 bp. **(B)** The normalized gene-body coverage of RNA-seq libraries was determined on the basis of *G. uralensis* AP and UP samples. For each library, the average coverage is presented for each relative position along the transcript.

Supplementary Figure S2 Distribution of different patterns of AS events in the licorice genome.

Supplementary Figure S3 Top 20 enriched molecular function GO terms among the DEGs under different drought conditions. Bar graphs present the percentage (%) of genes assigned specific GO terms.

Supplementary Figure S4 Cluster analysis of the differential expression of SPF-related genes after various drought treatments. Gene expression levels are indicated in terms of the transcripts per million (TPM) value. Upregulated and downregulated SPF-related genes are respectively indicated in yellow and blue in the heat map.

Supplementary Figure S5 AS profiles of SRF genes under APDS and UPDS conditions. **(A)** Glyur000842s00023837 (serine/arginine-rich splicing factor RS2Z33). **(B)** Glyur000959s00024505 (protein RDM16). **(C)** Glyur000404s00017726 (Sm-like protein LSM2). **(D)** Glyur000784s00031233 (phosphatidylinositol-glycan biosynthesis class F protein). Bar graphs present the relative expression levels of selectively spliced isoforms 1 (blue) and 2 (orange) under various drought stress conditions as determined by the RNA-seq analysis. The number in each blue/orange bar represents the proportion of the expression corresponding to isoforms 1/2.

Supplementary Figure S6 AS profiles of stress-responsive genes under APDS and UPDS conditions. **(A)** Glyur001192s00036935 (18.1 kDa class I heat shock protein). **(B)** Glyur000490s00023923 (22 kDa heat shock protein). **(C)** Glyur002052s00038821 (probable WRKY transcription factor 19). **(D)** Glyur000092s00007537 (probable WRKY transcription factor 20). **(E)** Glyur001392s00030472 (probable protein phosphatase 2C 76). **(F)** Glyur000061s00006499 (probable protein phosphatase 2C 6). **(G)** Glyur000105s00014180 (protein phosphatase 2C 16). **(H)** Glyur000521s00021495 (probable protein phosphatase 2C 26). Bar graphs present the relative expression levels of selectively spliced isoforms 1 (blue) and 2 (red) under various drought stress conditions as determined by the RNA-seq analysis. The number in each blue/red bar represents the proportion of the expression corresponding to isoforms 1/2.

Supplementary Figure S7 Relative expression of HSP22 **(A)**, WRKY19 **(B)**, WRKY20 **(C)**, and PP2C **(D)** in licorice under APDS and UPDS conditions. Data are presented as the mean ± standard deviation of three biological replicates. The mean values for different time points under drought stress were analyzed using the Bonferroni test and T-test. Different letters indicate highly significant differences among treatments. *: *p* < 0.05, **: *p* < 0.01, a: *p* < 0.05, A: *p* < 0.01.
