# Supplementary material for: Analysis of Whole-Transcriptome RNA-Seq Data Reveals the Involvement of Alternative Splicing in the Drought Response of Glycyrrhiza uralensis
Source: Front Genet. 2022 May 17;13:885651. doi: 10.3389/fgene.2022.885651 (PMC9152209; doi:10.3389/fgene.2022.885651)
Supplement: Supplementary file 1 [file DataSheet1.zip › Supplementary Figure SXXX/Supplementary Figure S5.pdf]

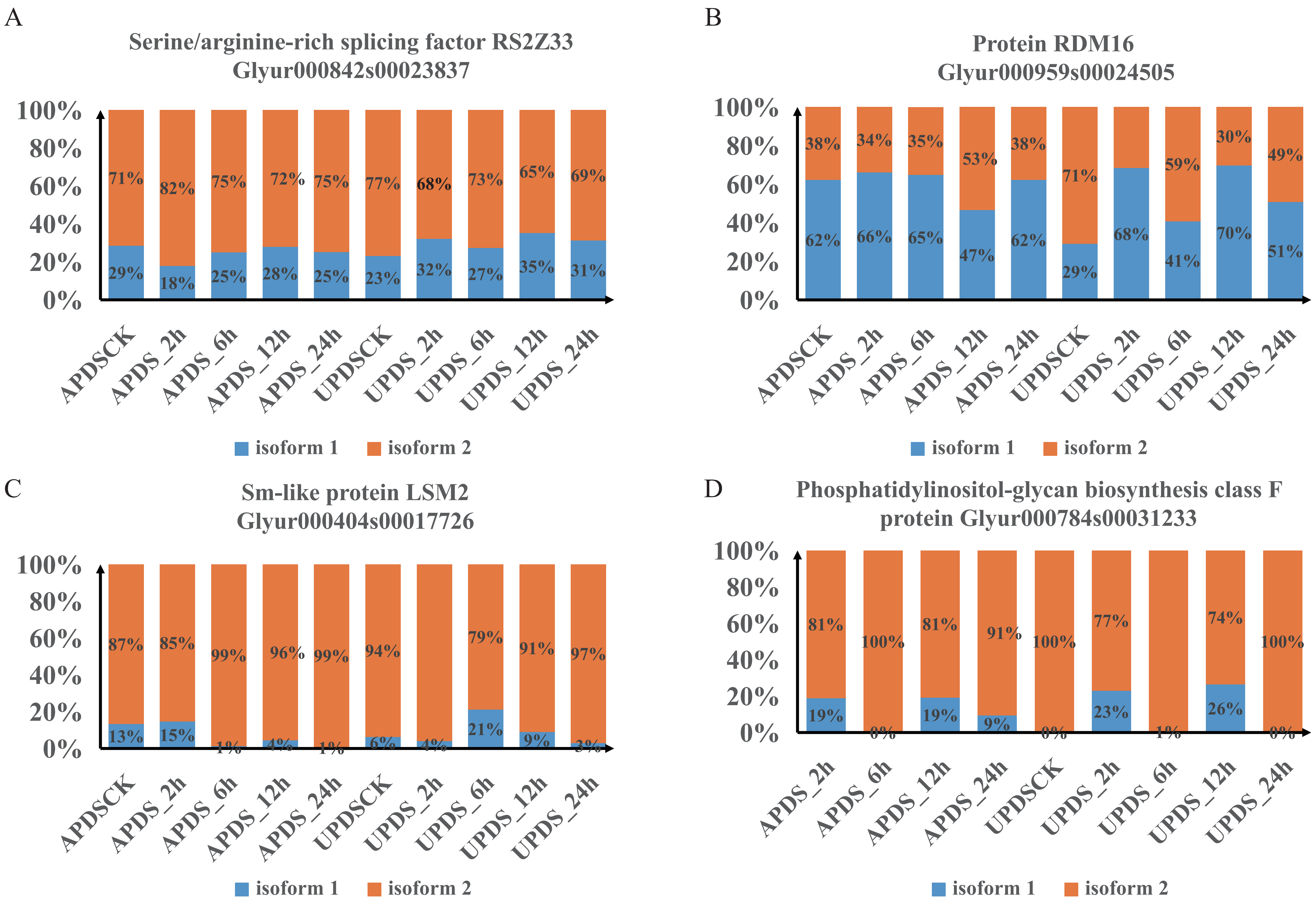
A
Serine/arginine-rich splicing factor RS2Z33
Glyur000842s00023837
100%
80%
60%
40%
20%
0%
71% 82% 75% 72% 75% 77% 68% 73% 65% 69%
29% 18% 25% 28% 25% 23% 32% 27% 35% 31%
APDSCK APDS_2h APDS_6h APDS_12h APDS_24h UPDSCK UPDS_2h UPDS_6h UPDS_12h UPDS_24h
isoform 1
isoform 2
B
Protein RDM16
Glyur000959s00024505
100%
80%
60%
40%
20%
0%
38% 34% 35% 53% 38% 71% 59% 30% 49%
62% 66% 65% 47% 62% 29% 68% 41% 70% 51%
APDSCK APDS_2h APDS_6h APDS_12h APDS_24h UPDSCK UPDS_2h UPDS_6h UPDS_12h UPDS_24h
isoform 1
isoform 2
C
Sm-like protein LSM2
Glyur000404s00017726
100%
80%
60%
40%
20%
0%
87% 85% 99% 96% 99% 94% 79% 91% 97%
13% 15% 1% 4% 1% 6% 4% 21% 9% 3%
APDSCK APDS_2h APDS_6h APDS_12h APDS_24h UPDSCK UPDS_2h UPDS_6h UPDS_12h UPDS_24h
isoform 1
isoform 2
D
Phosphatidylinositol-glycan biosynthesis class F
protein Glyur000784s00031233
100%
80%
60%
40%
20%
0%
81% 100% 81% 91% 100% 77% 74% 100%
19% 0% 19% 9% 0% 23% 1% 26% 0%
APDS_2h APDS_6h APDS_12h APDS_24h UPDSCK UPDS_2h UPDS_6h UPDS_12h UPDS_24h
isoform 1
isoform 2
