# Supplementary figures and images for: Analysis of Whole-Transcriptome RNA-Seq Data Reveals the Involvement of Alternative Splicing in the Drought Response of Glycyrrhiza uralensis
Source: Front Genet. 2022 May 17;13:885651. doi: 10.3389/fgene.2022.885651 (PMC9152209; doi:10.3389/fgene.2022.885651)

A

## Reads distribution in chromosomes

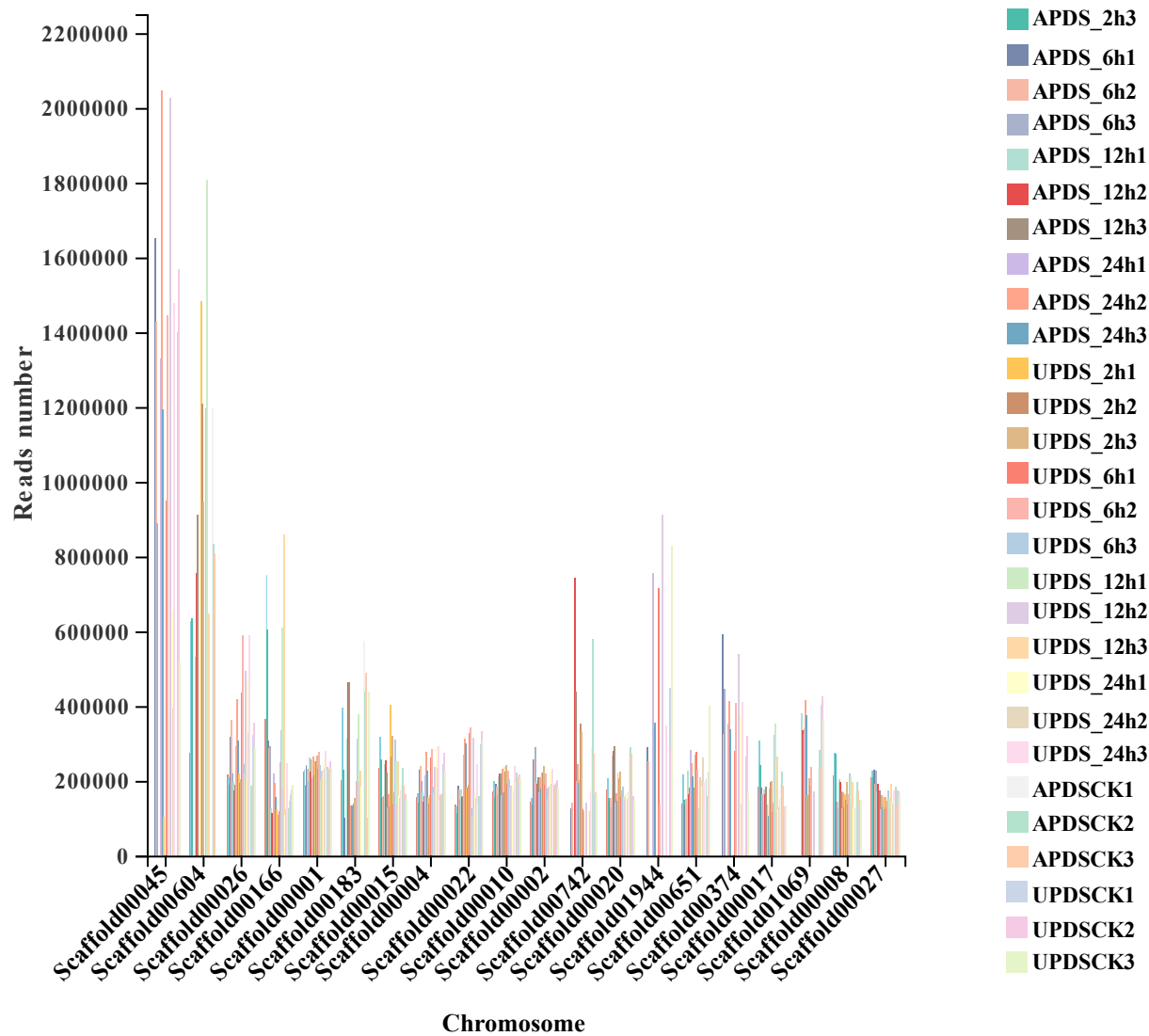

## Coverage of sequencing

B

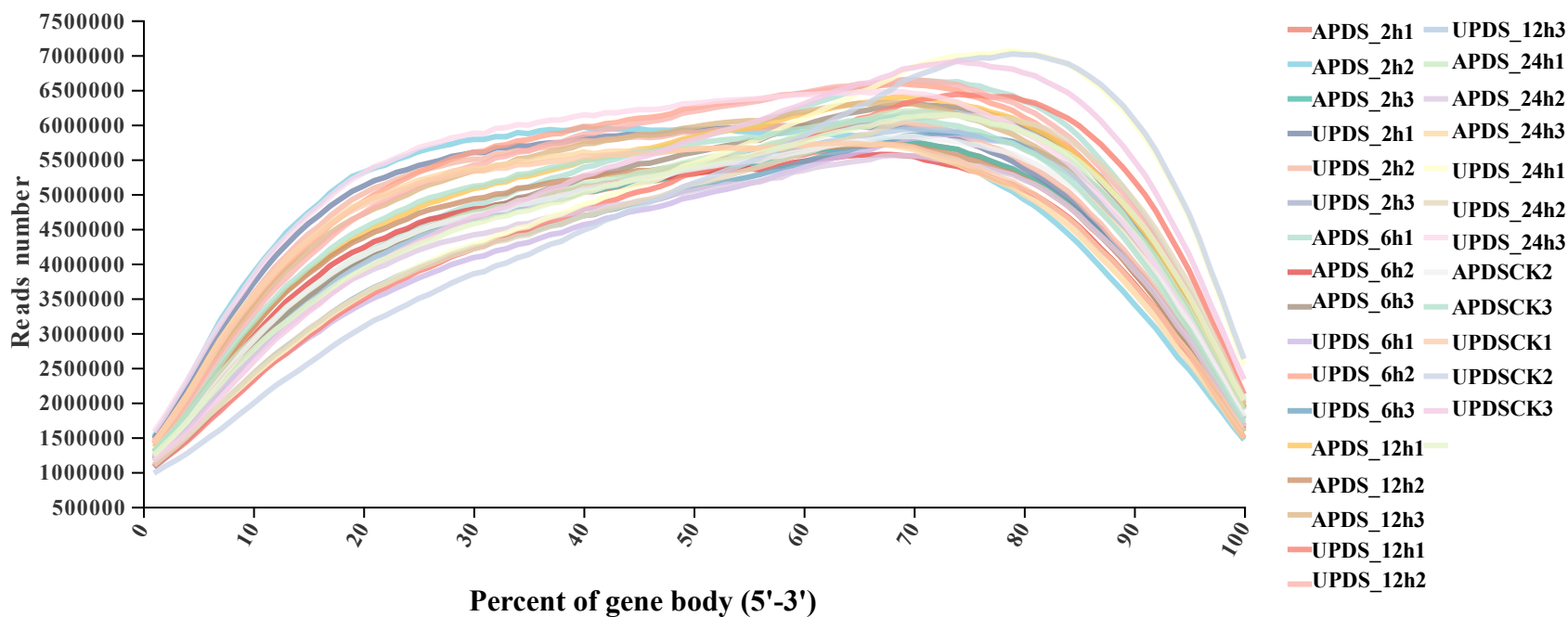

Supplement: Supplementary file 1 [file DataSheet1.zip › Supplementary Figure SXXX/Supplementary Figure S1.pdf]

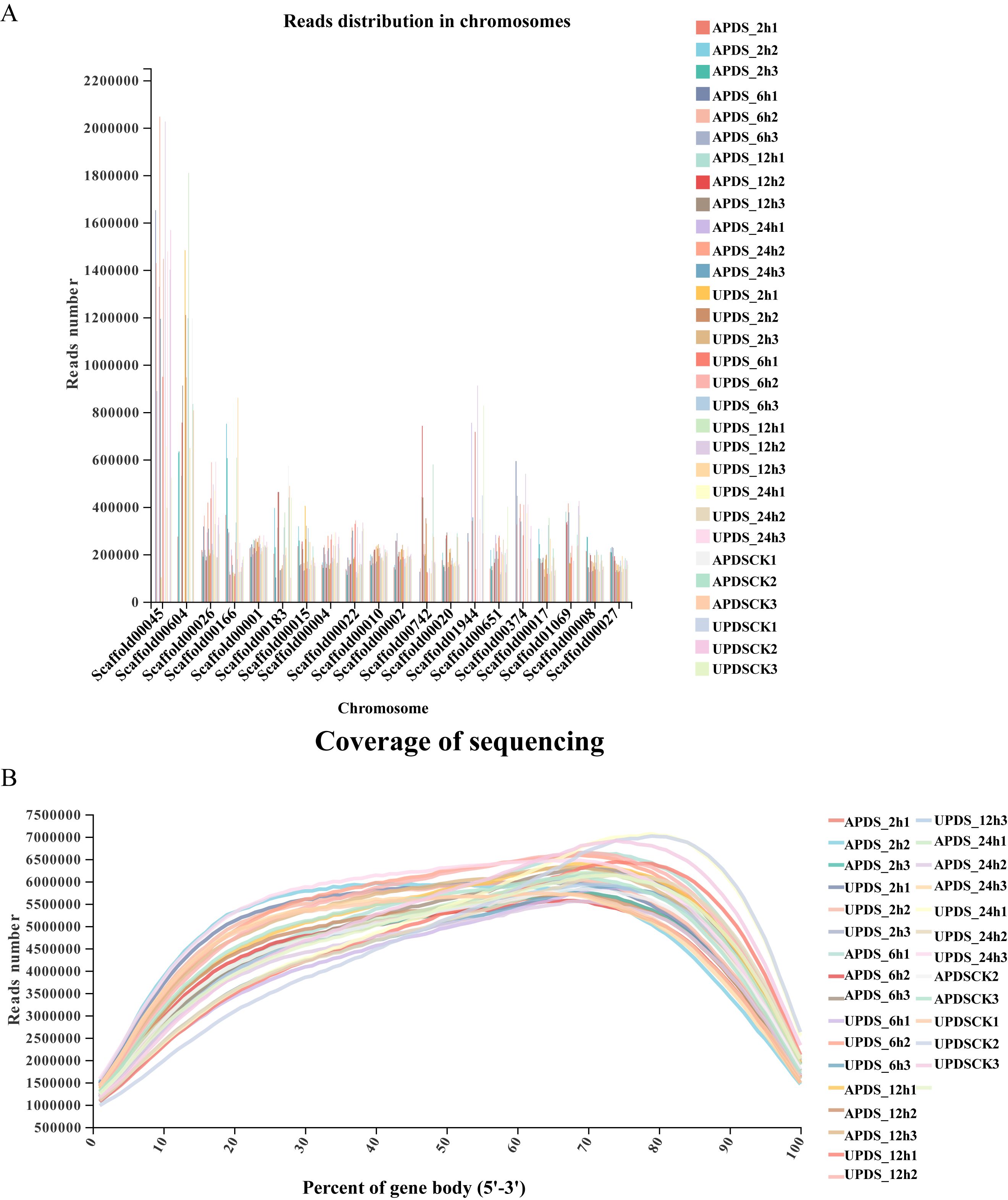

Supplement: Supplementary file 1 [file DataSheet1.zip › Supplementary Figure SXXX/Supplementary Figure S1.tif]

A

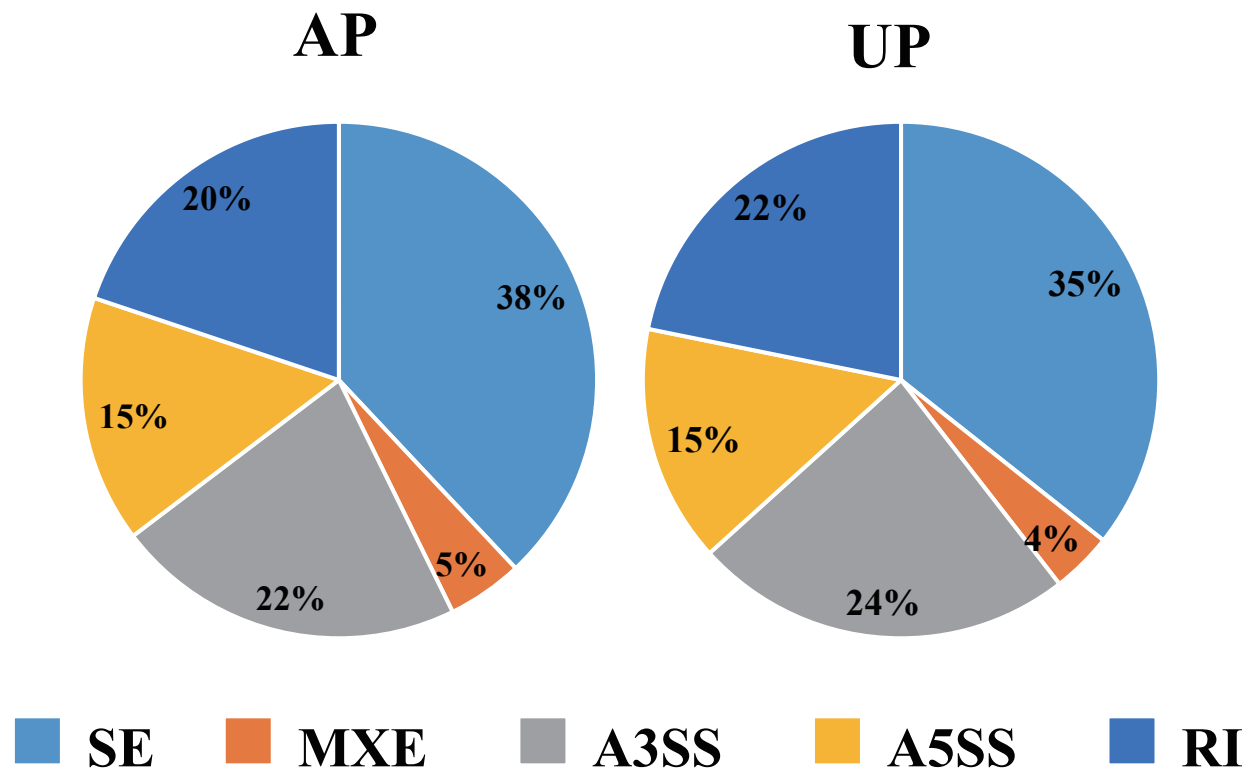

B

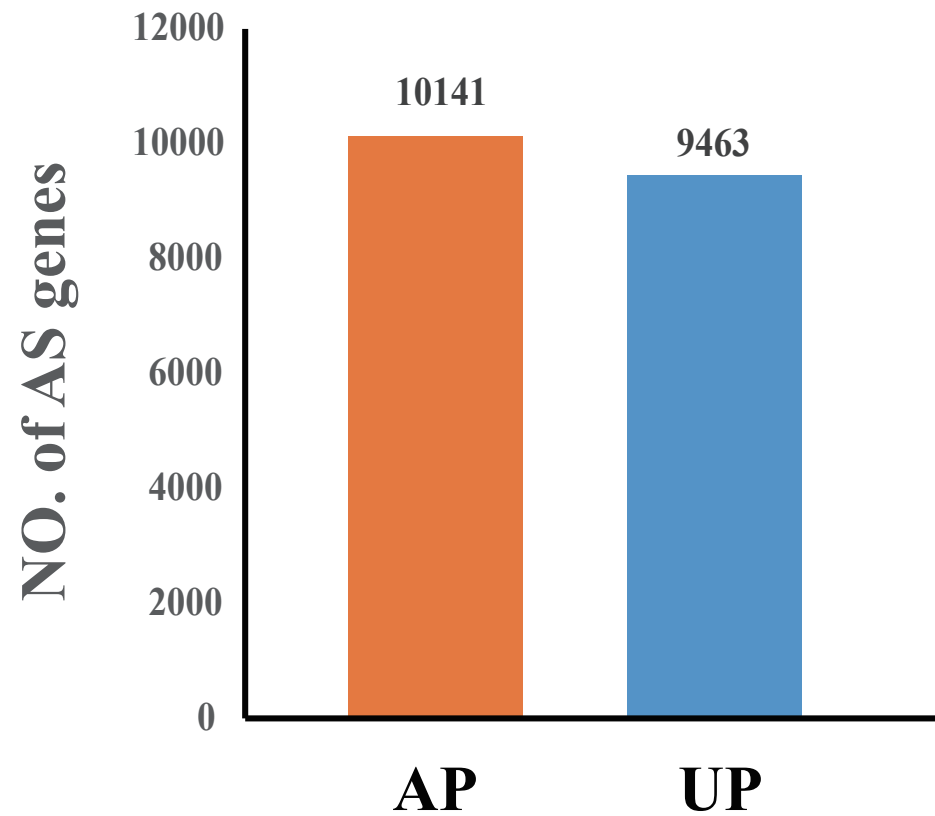

Supplement: Supplementary file 1 [file DataSheet1.zip › Supplementary Figure SXXX/Supplementary Figure S2.pdf]

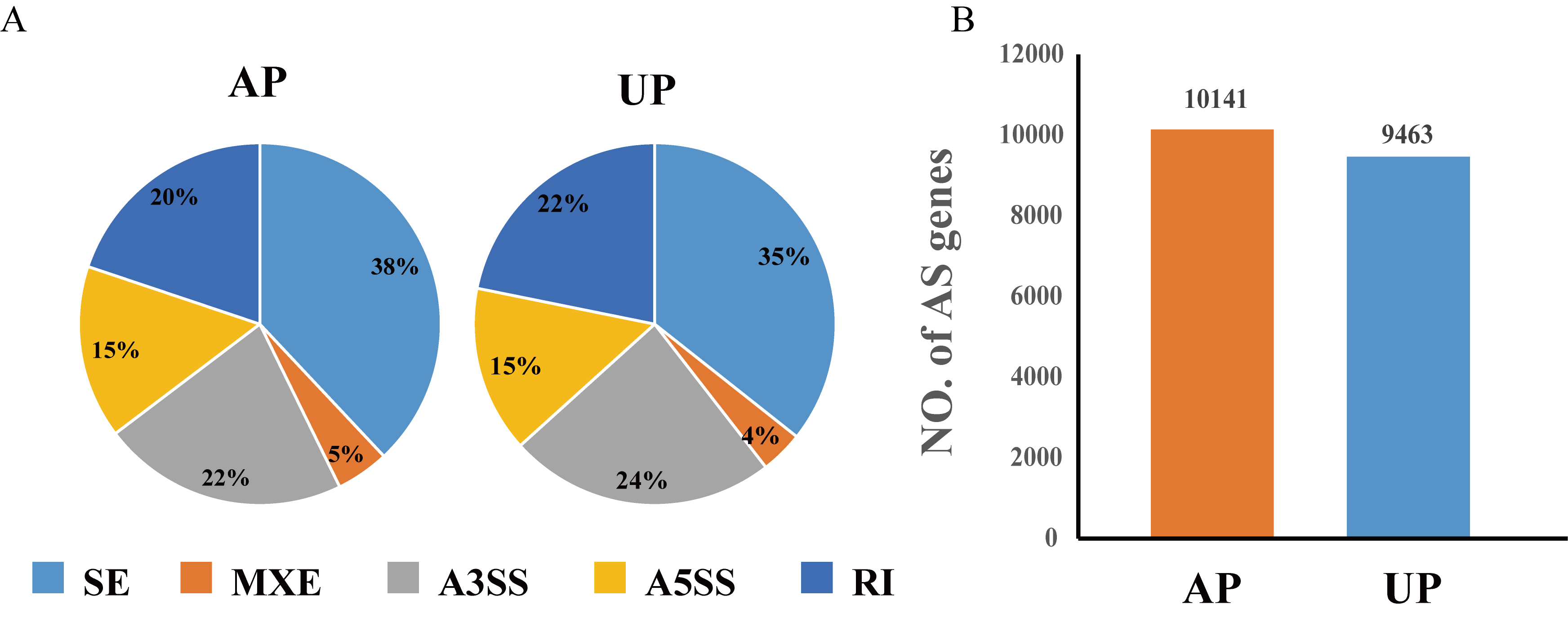

Supplement: Supplementary file 1 [file DataSheet1.zip › Supplementary Figure SXXX/Supplementary Figure S2.tif]

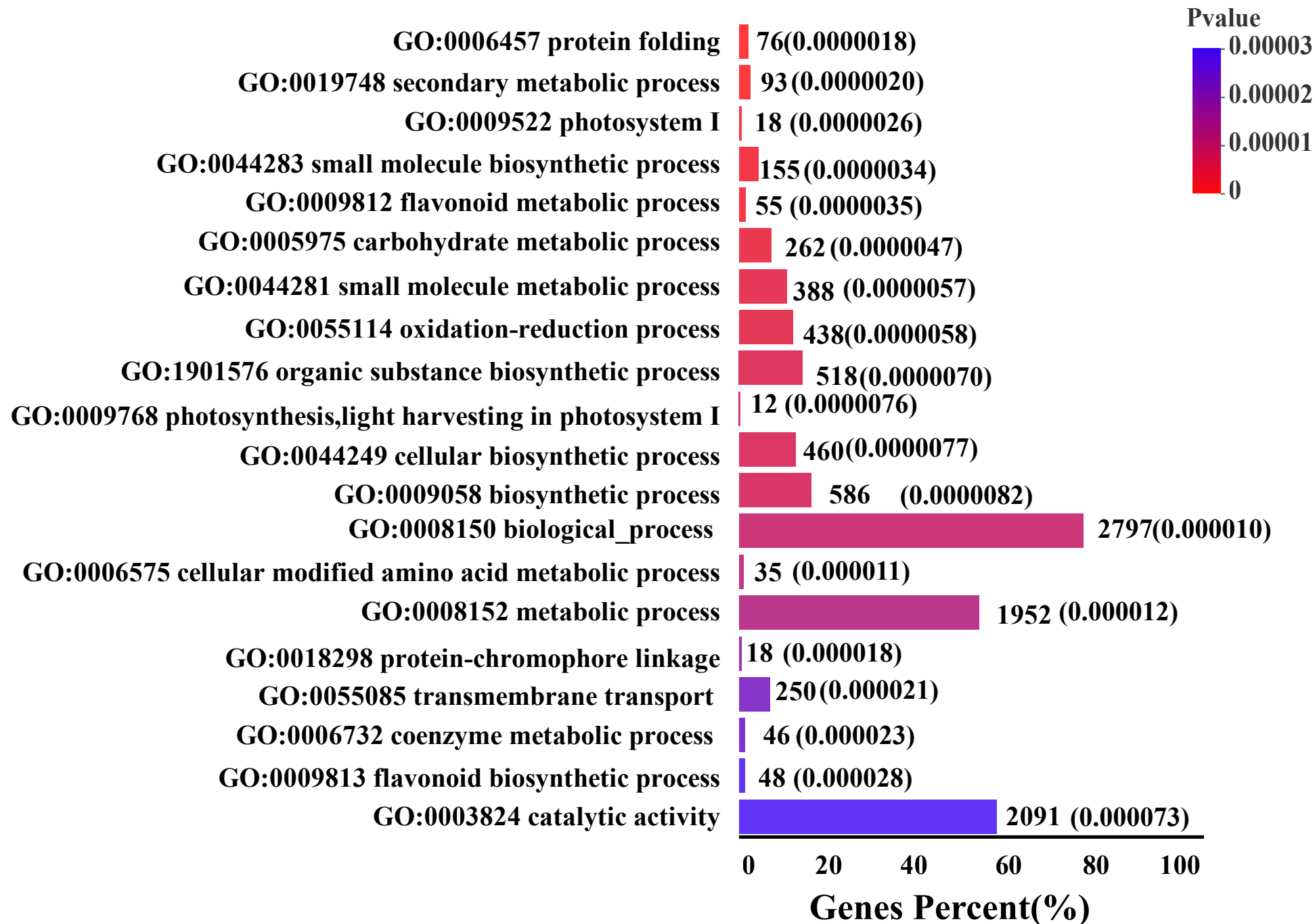

Supplement: Supplementary file 1 [file DataSheet1.zip › Supplementary Figure SXXX/Supplementary Figure S3.pdf]

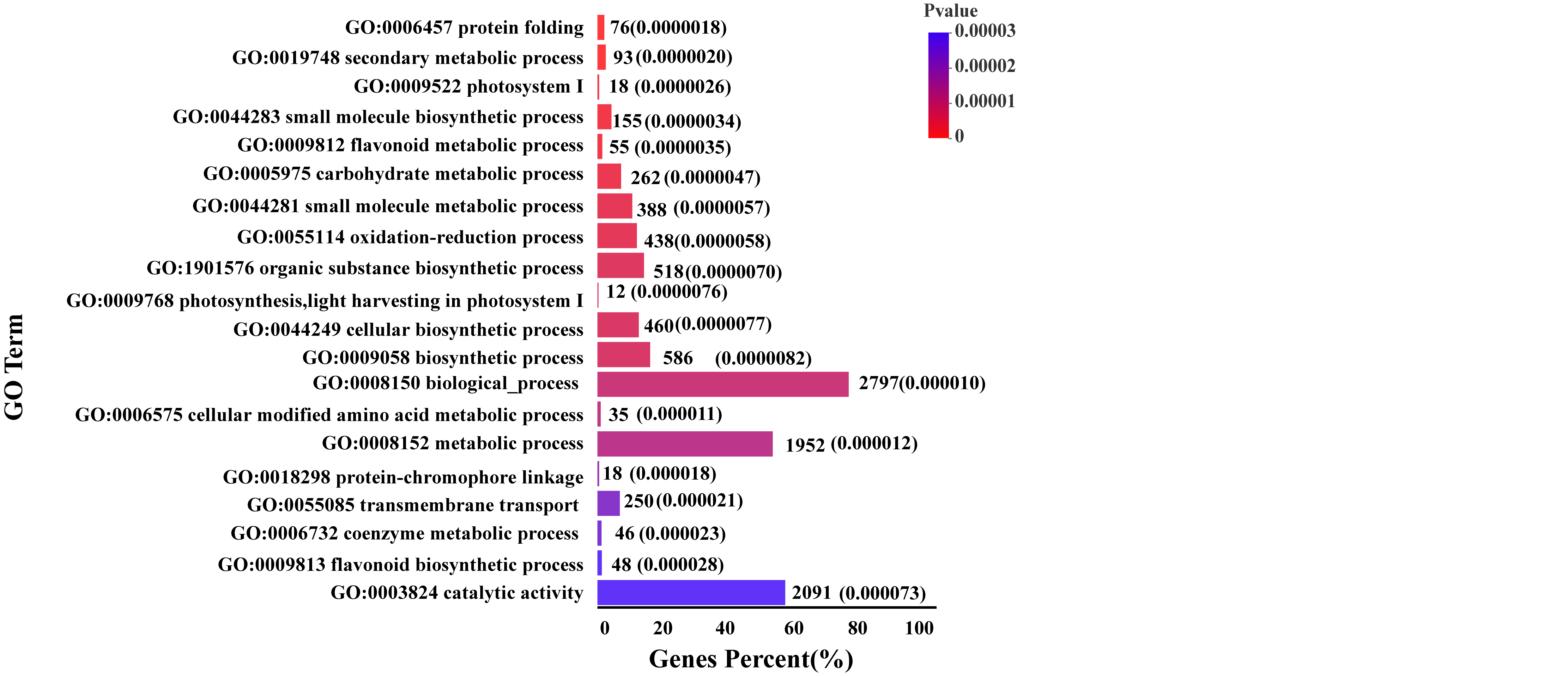

Supplement: Supplementary file 1 [file DataSheet1.zip › Supplementary Figure SXXX/Supplementary Figure S3.tif]

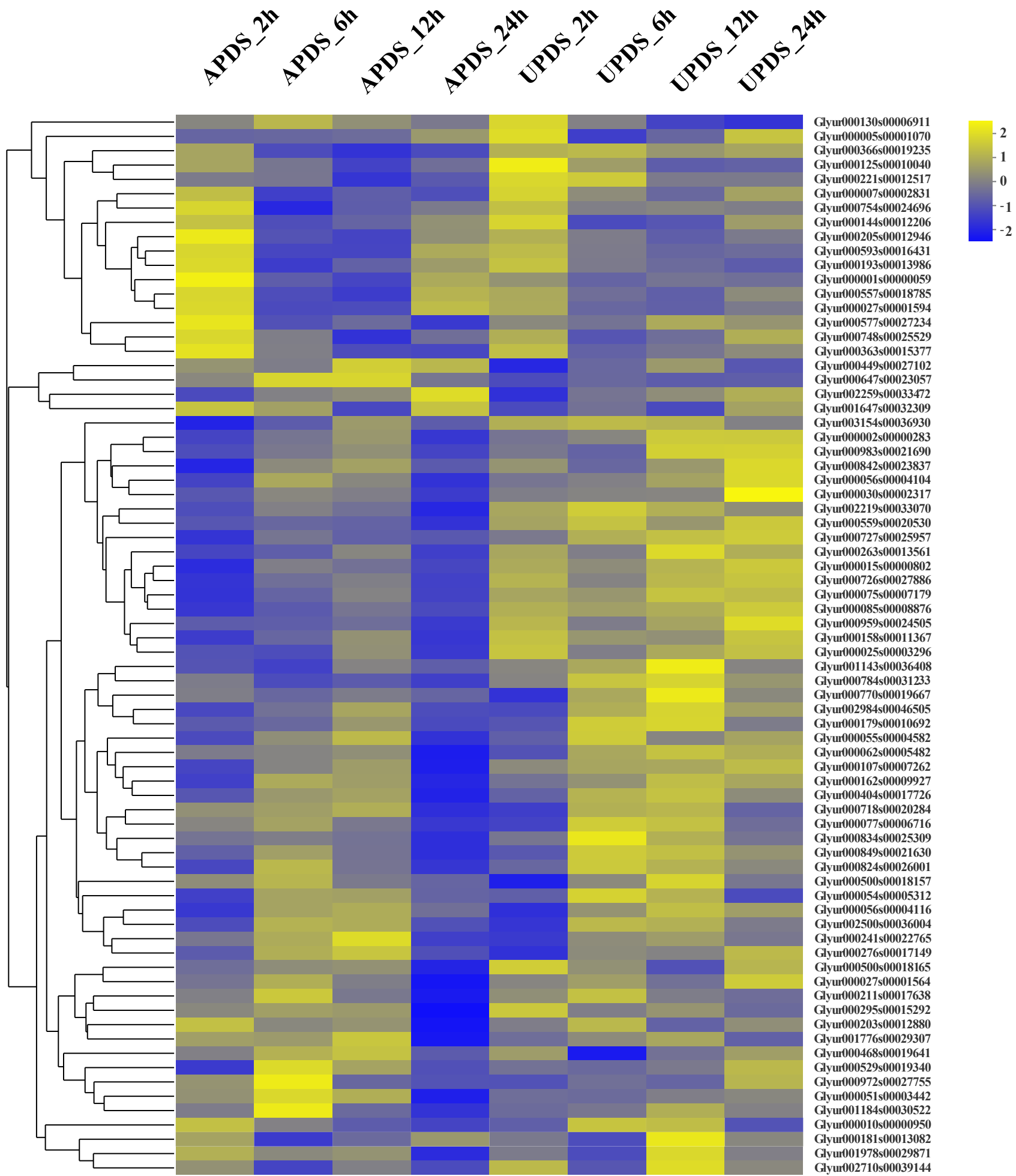

Supplement: Supplementary file 1 [file DataSheet1.zip › Supplementary Figure SXXX/Supplementary Figure S4.pdf]

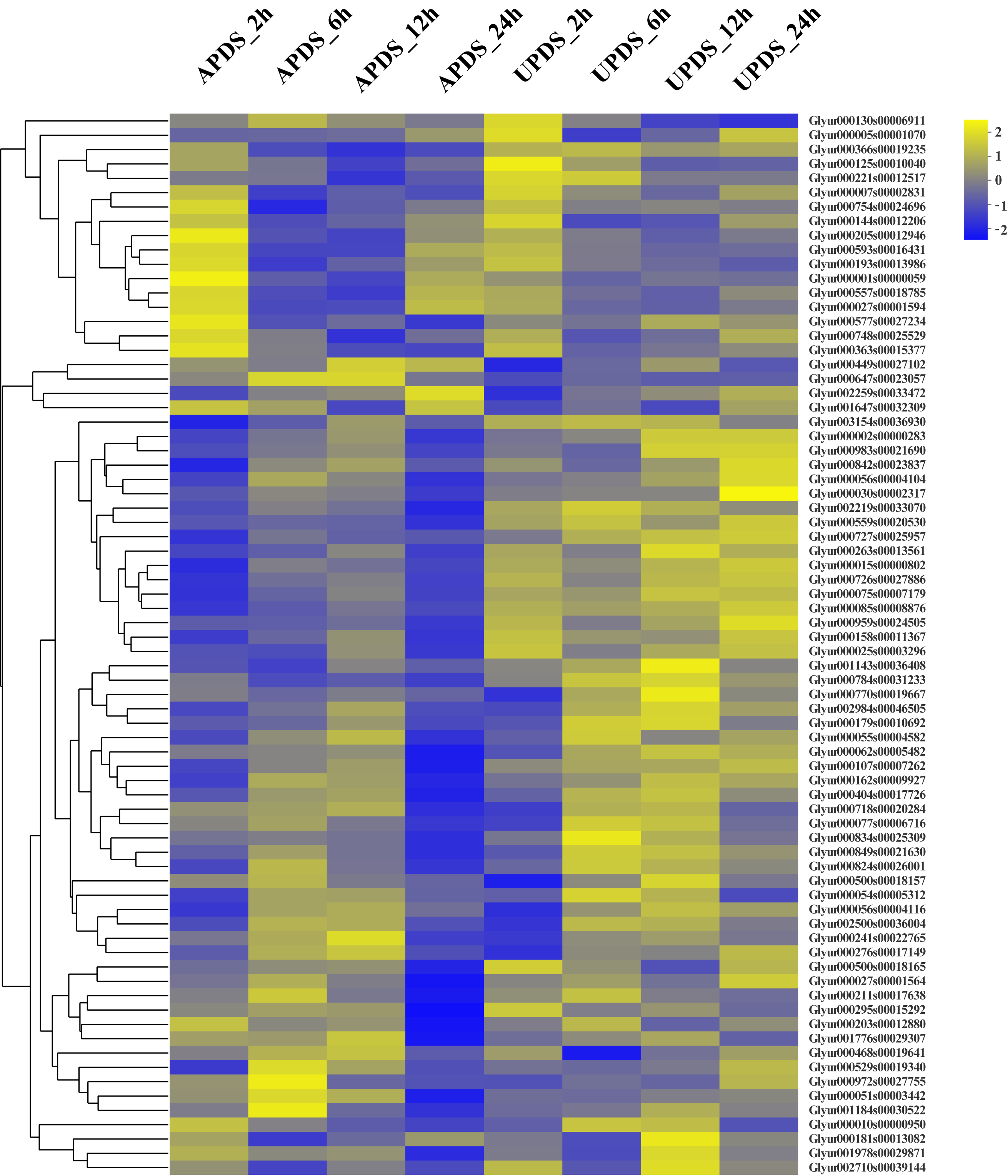

Supplement: Supplementary file 1 [file DataSheet1.zip › Supplementary Figure SXXX/Supplementary Figure S4.tif]

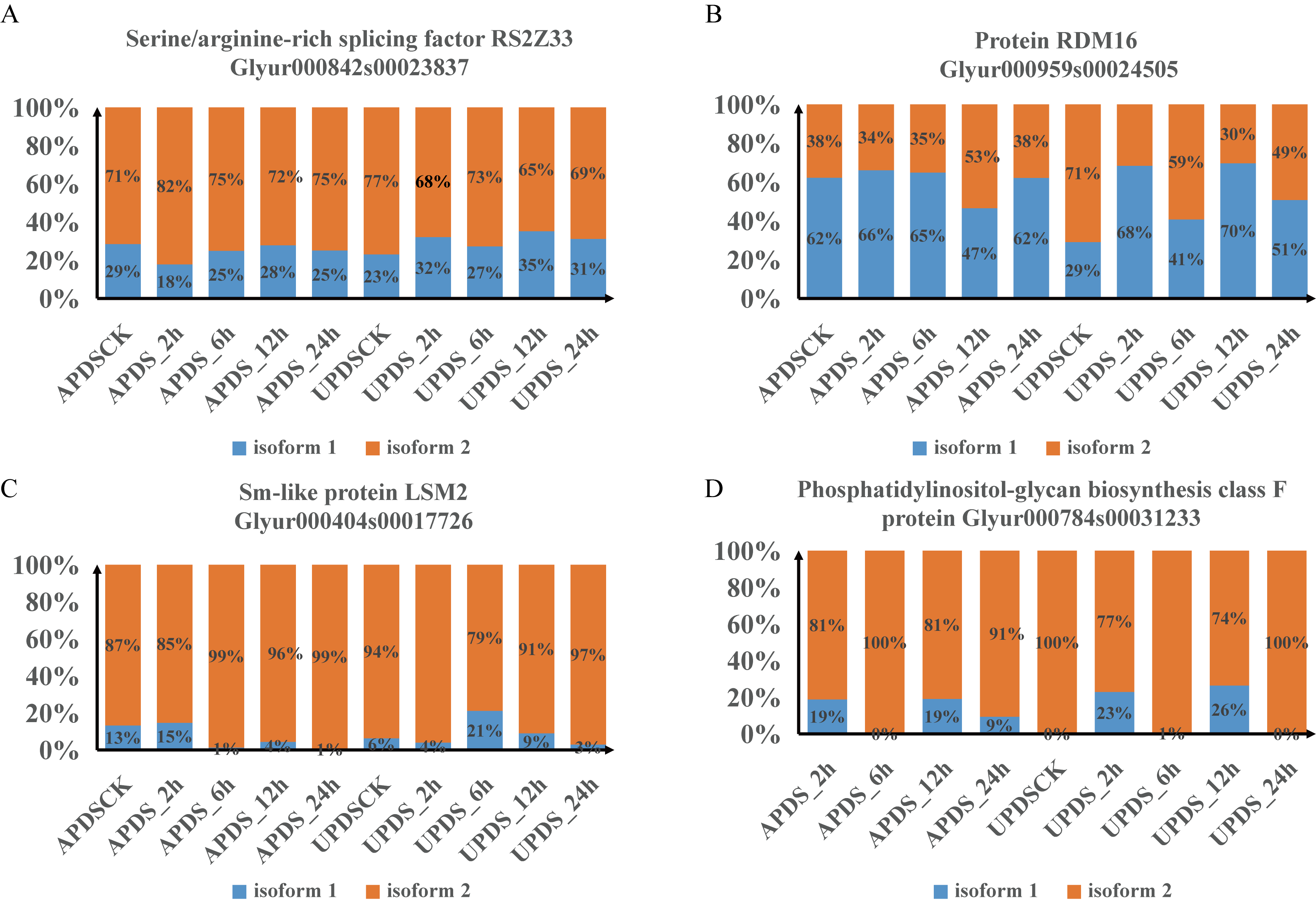

Supplement: Supplementary file 1 [file DataSheet1.zip › Supplementary Figure SXXX/Supplementary Figure S5.tif]

A

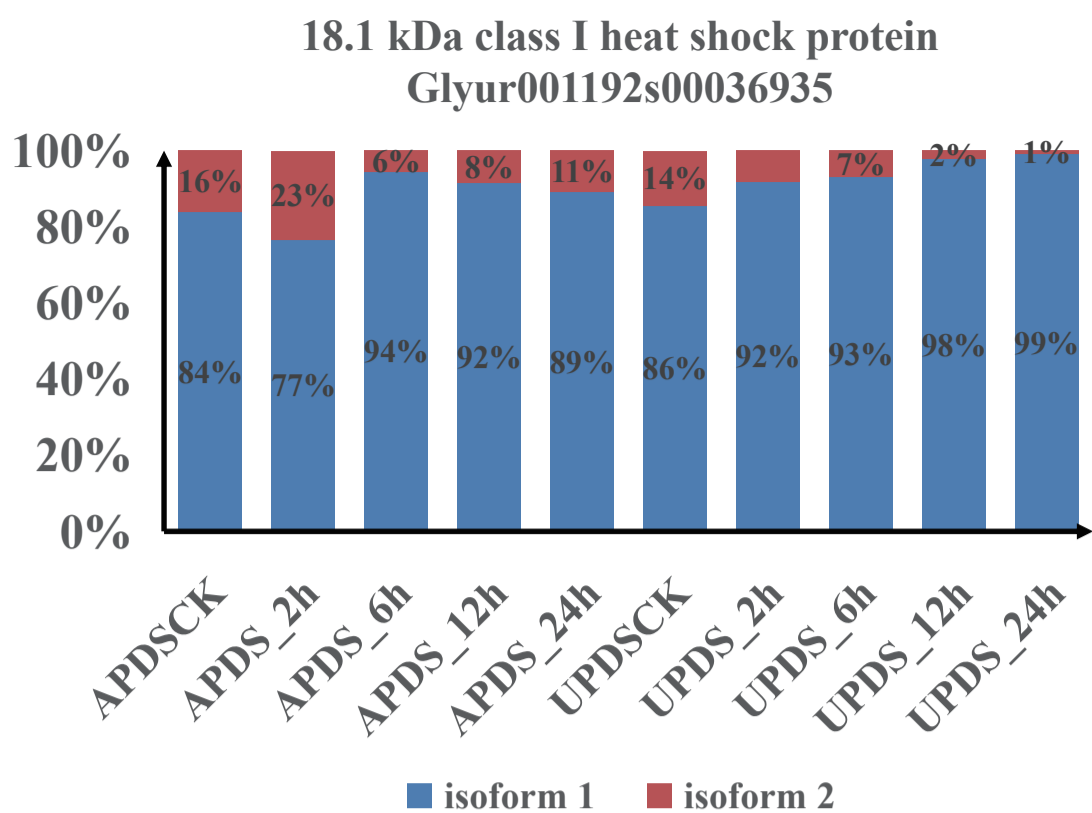

B

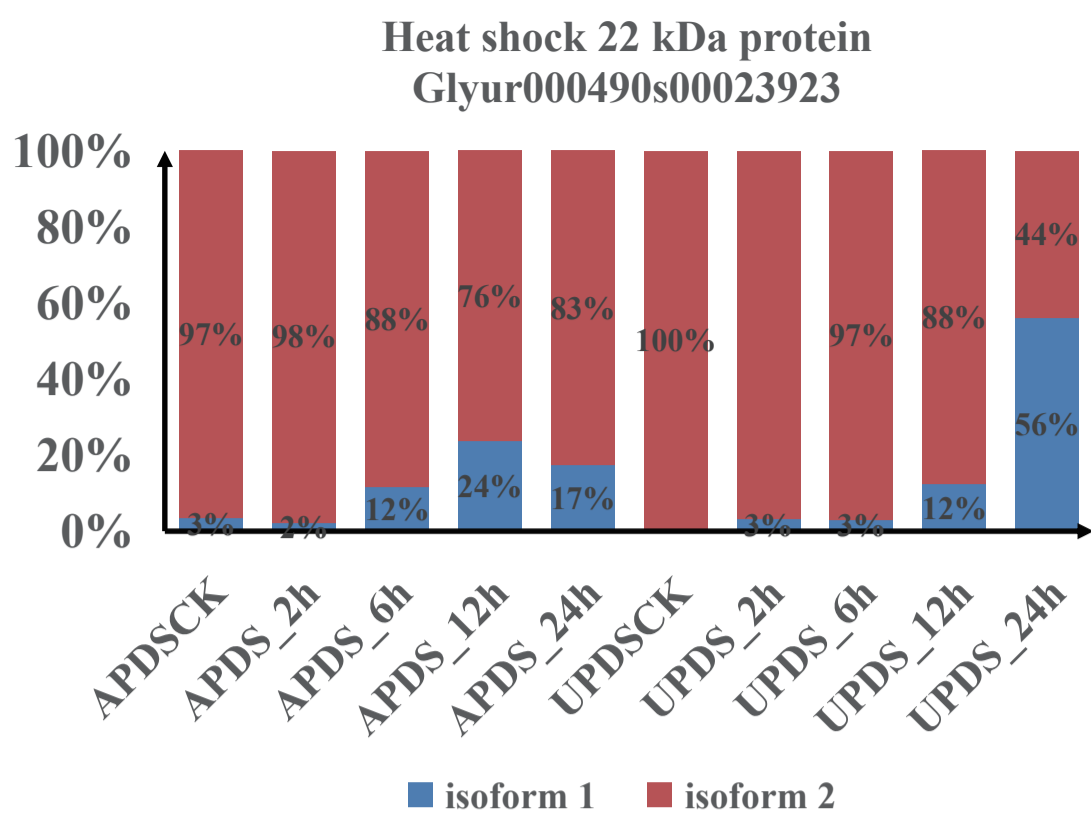

C

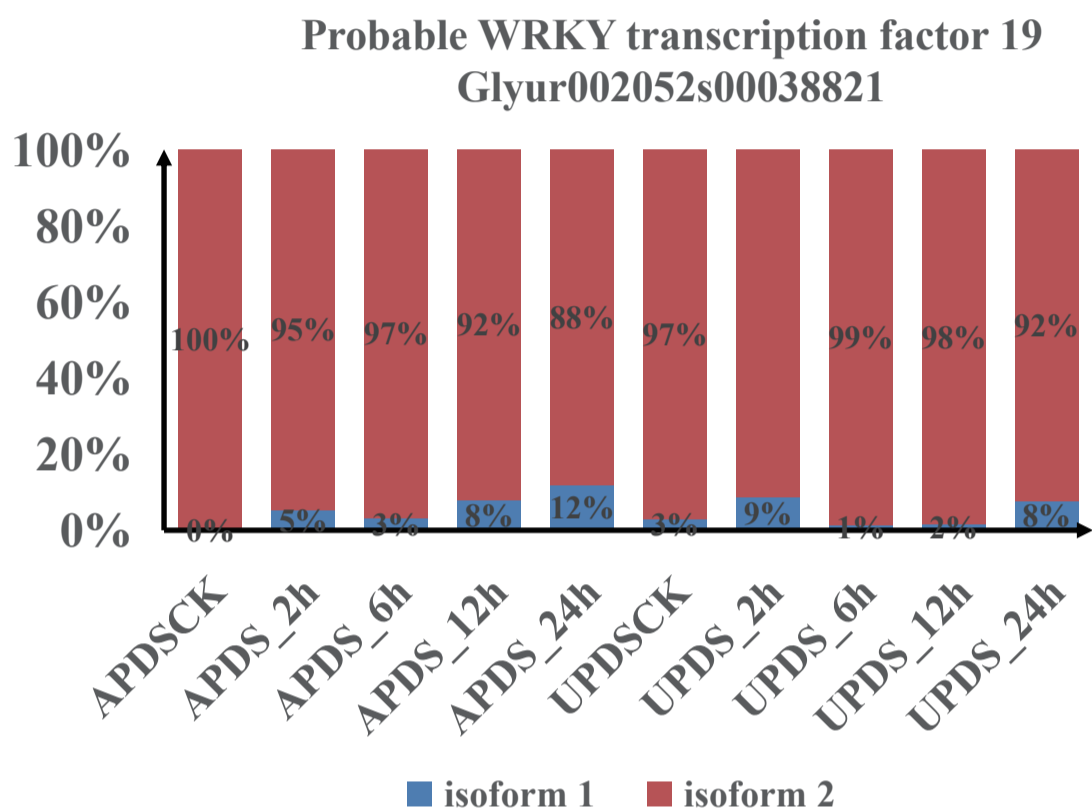

D

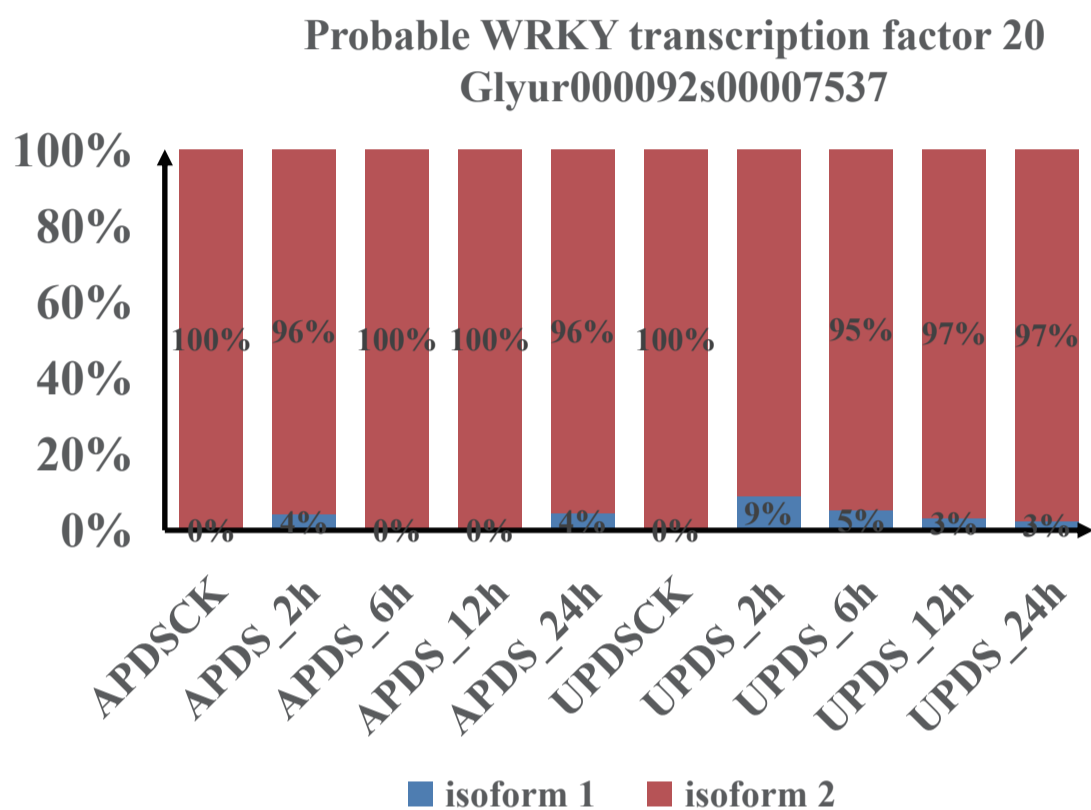

E

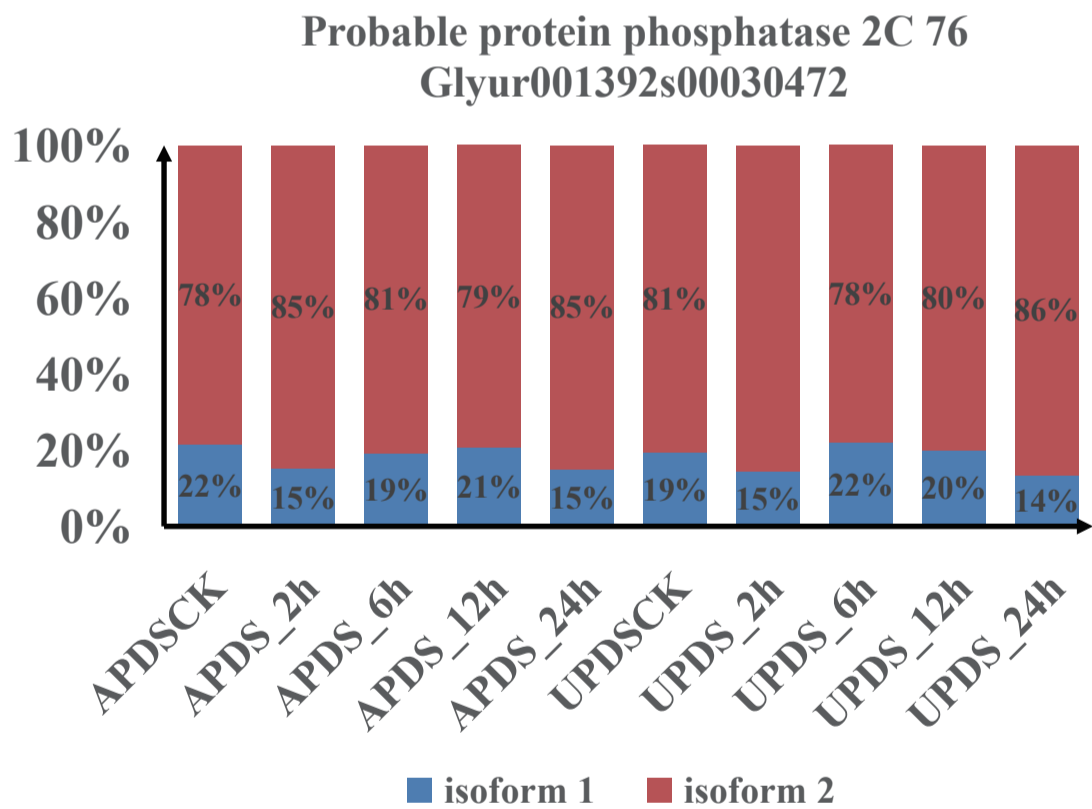

F

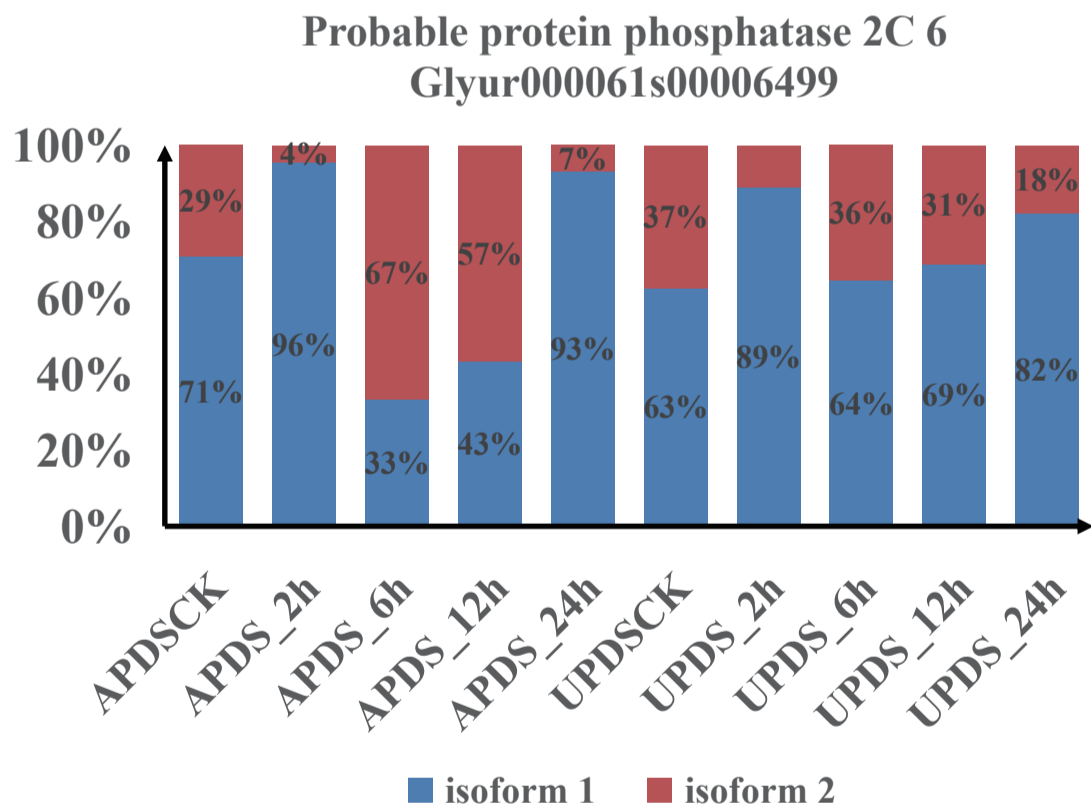

G

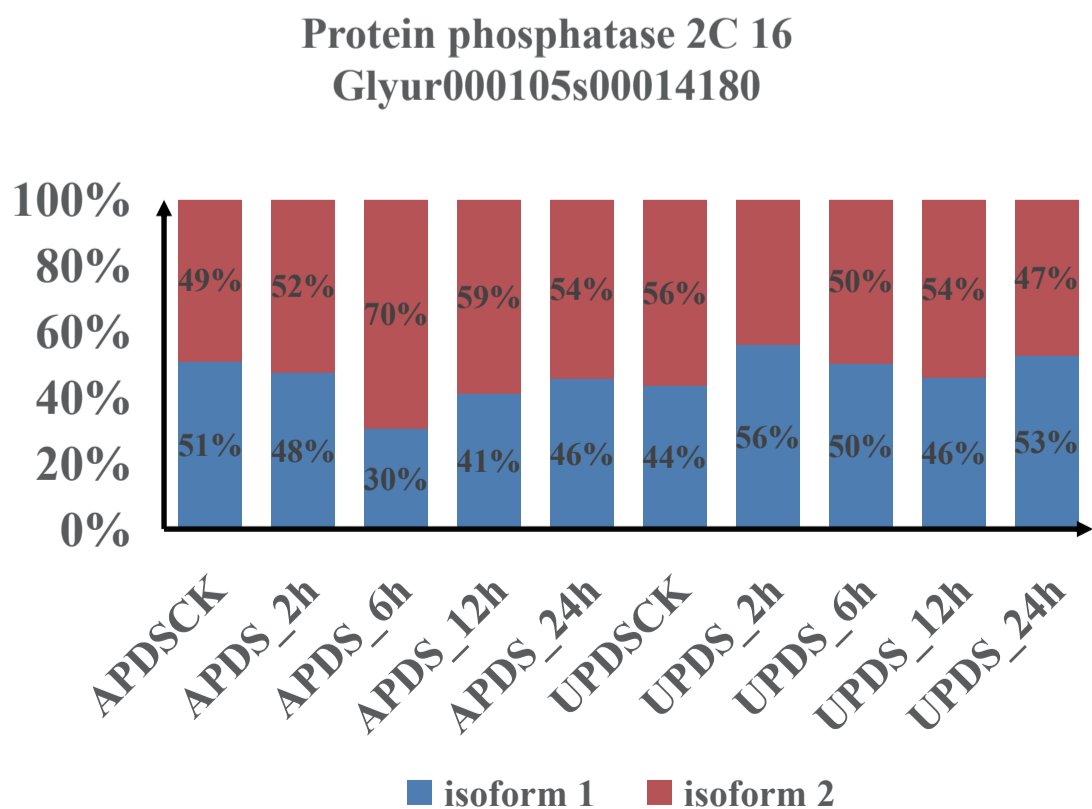

H

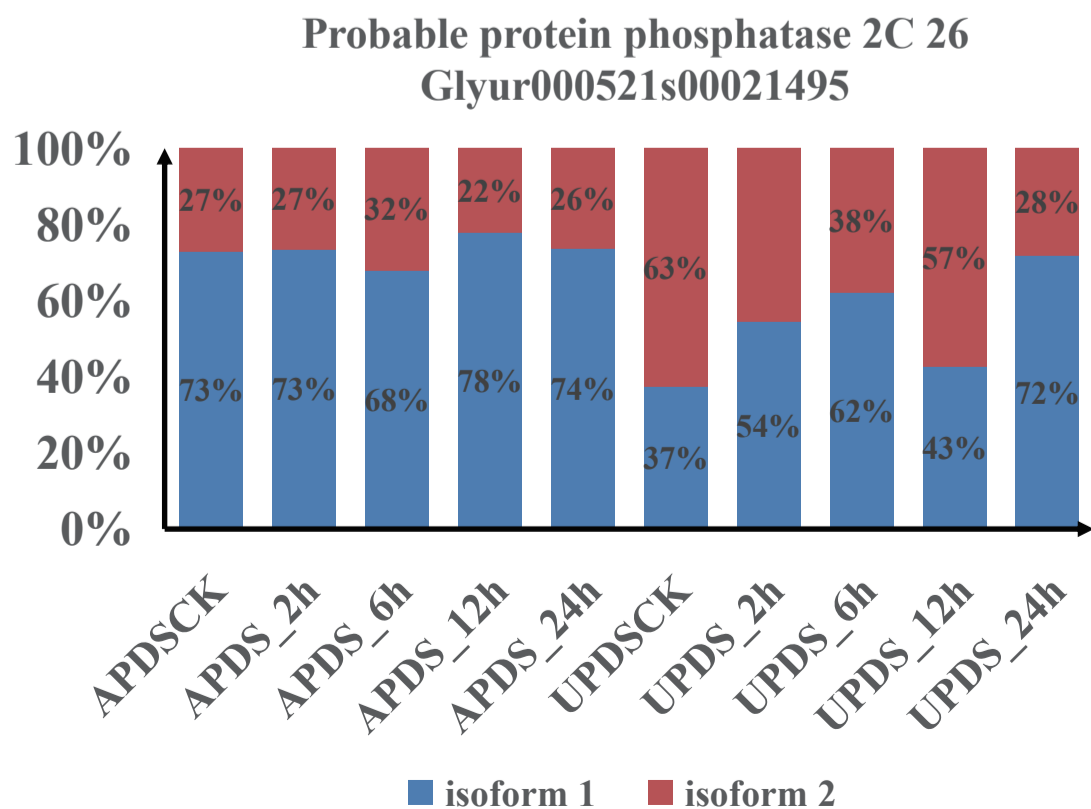

Supplement: Supplementary file 1 [file DataSheet1.zip › Supplementary Figure SXXX/Supplementary Figure S6.pdf]

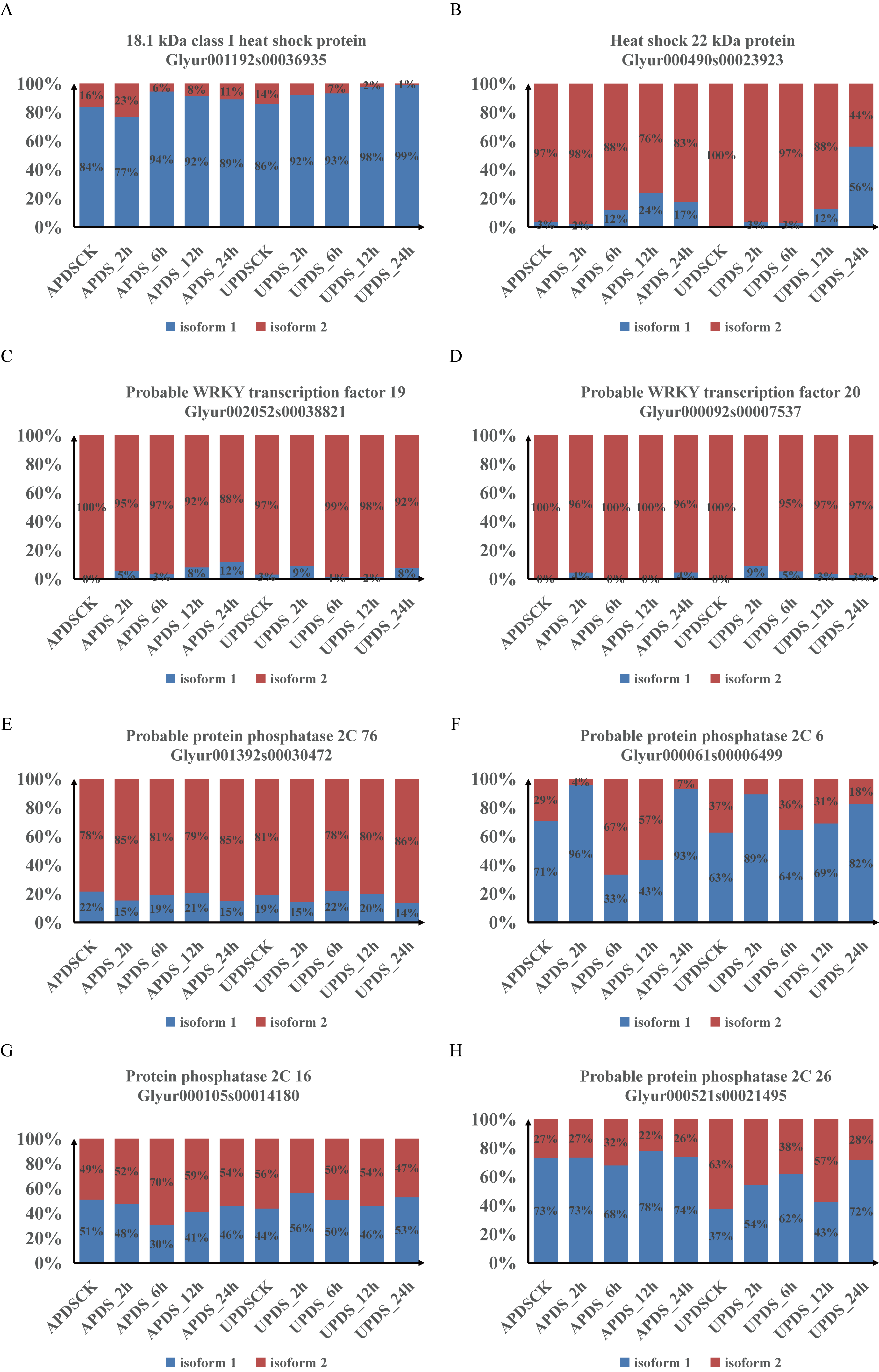

Supplement: Supplementary file 1 [file DataSheet1.zip › Supplementary Figure SXXX/Supplementary Figure S6.tif]

CK 2h 6h 12h 24h

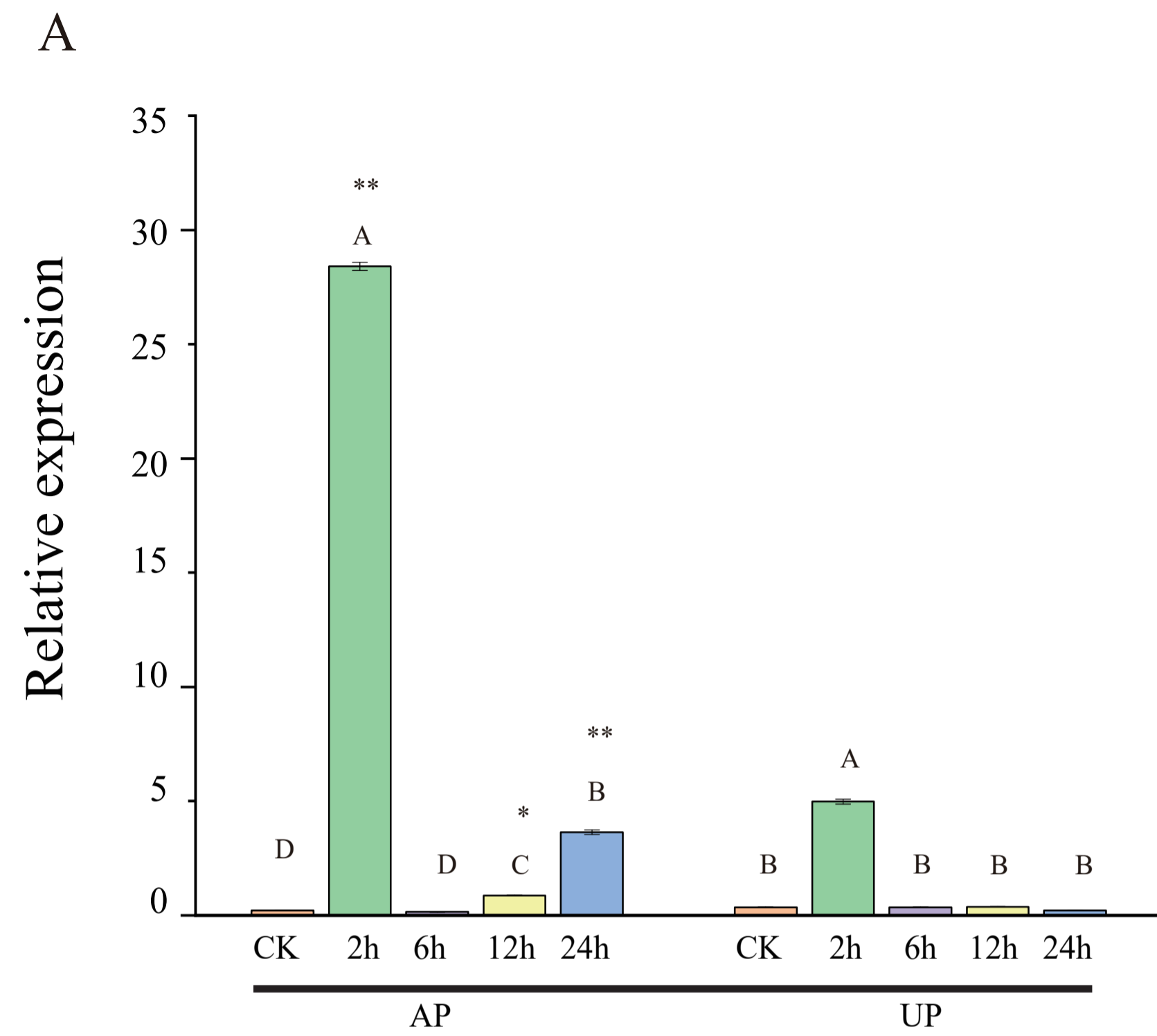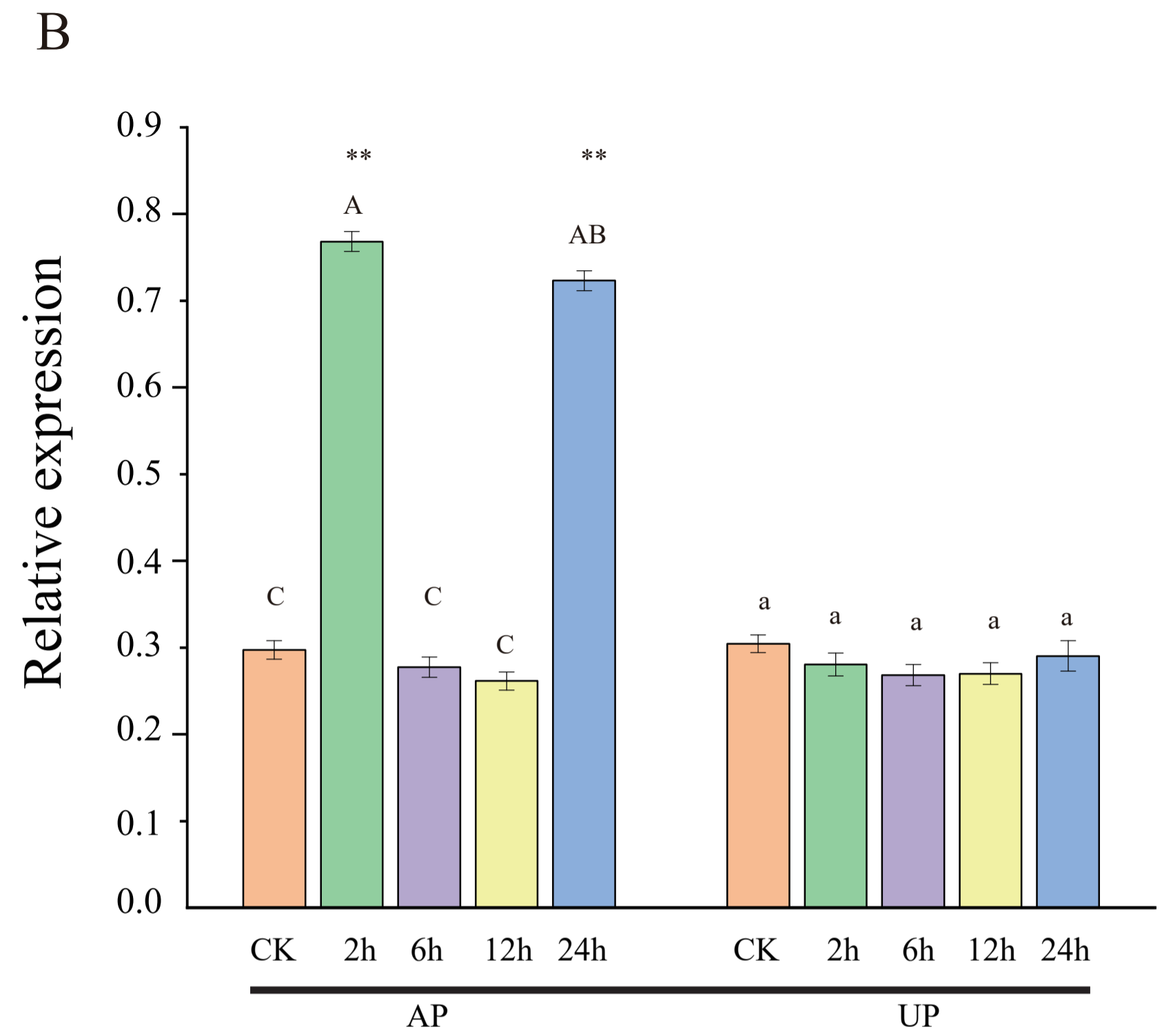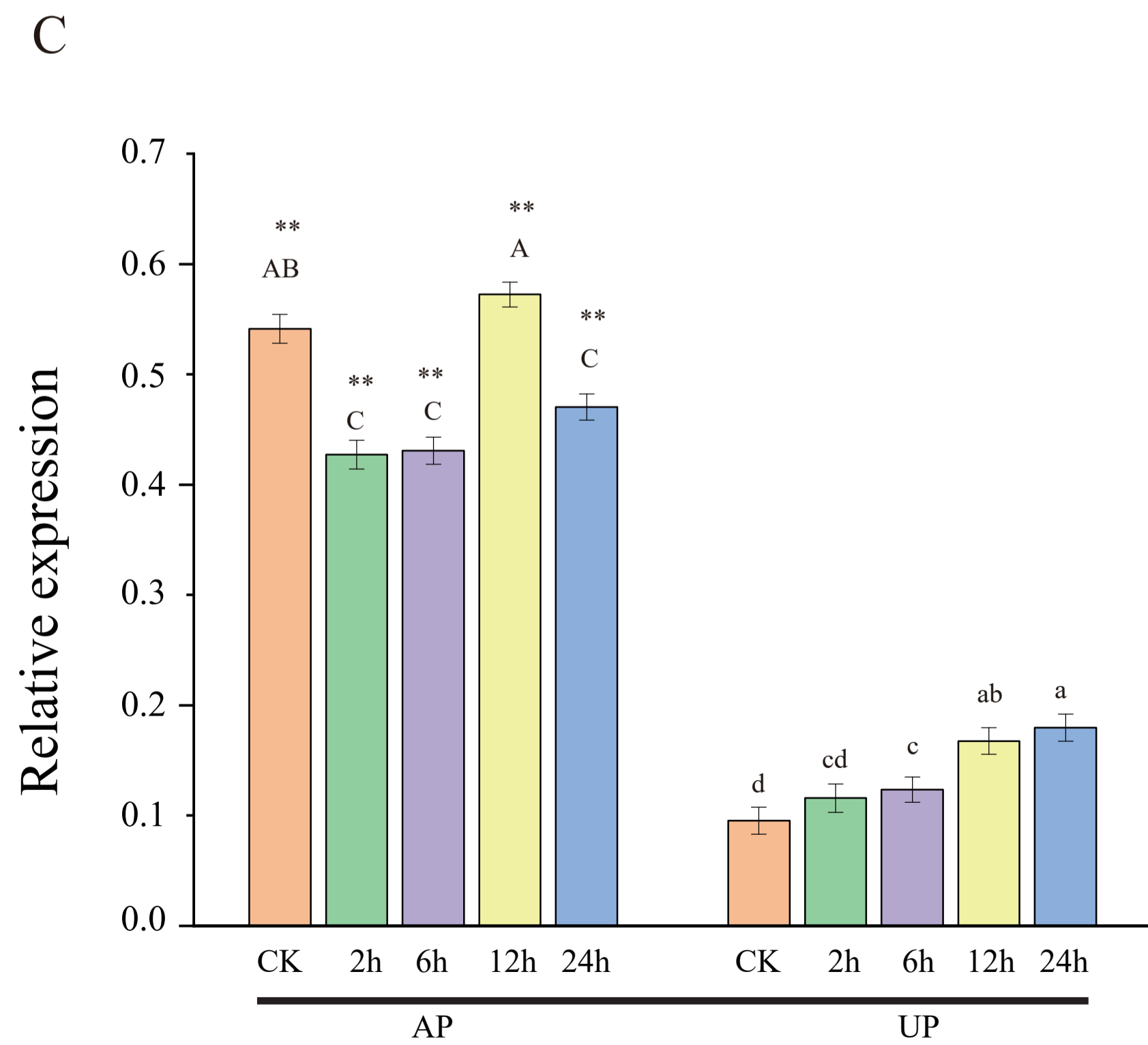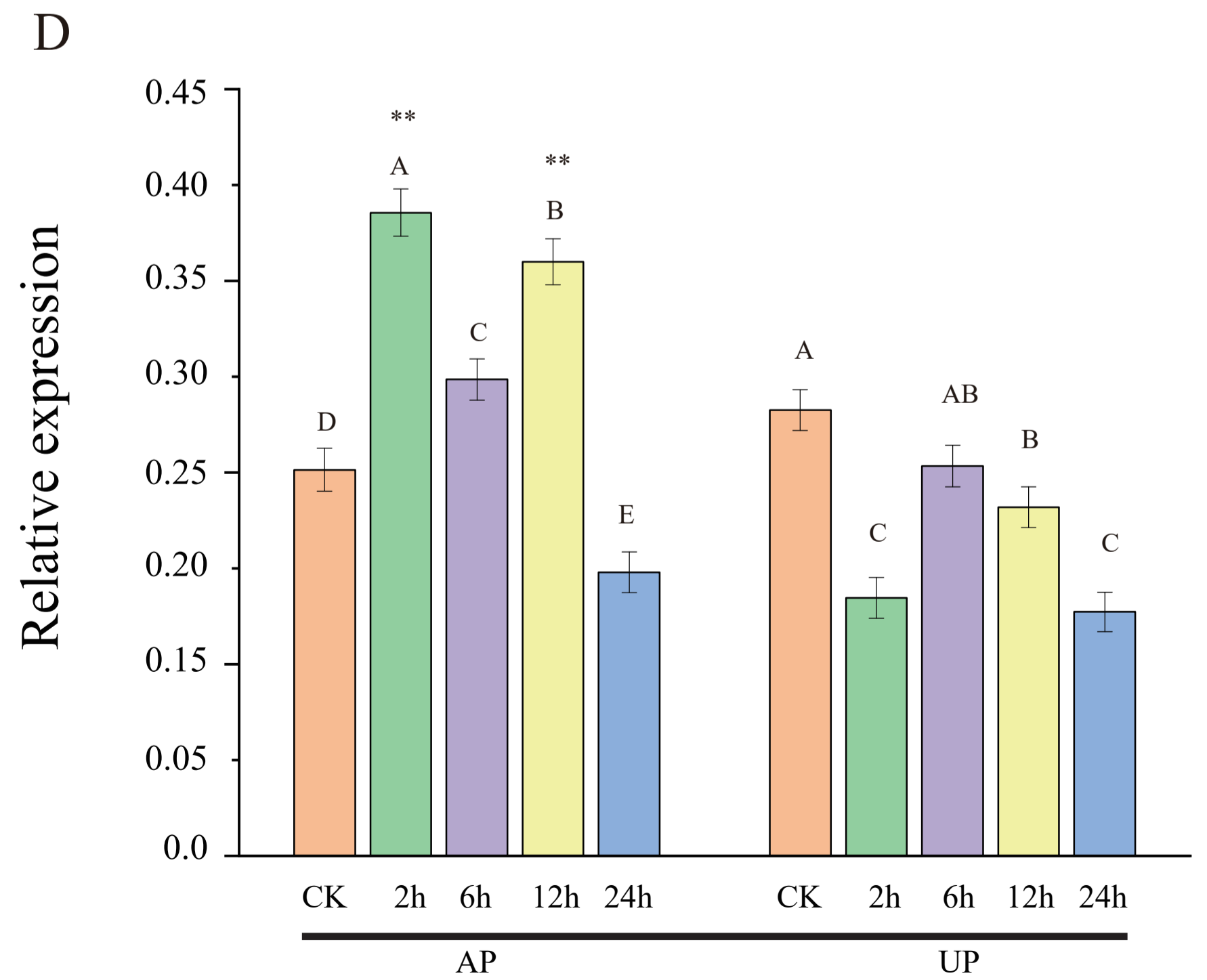

Supplement: Supplementary file 1 [file DataSheet1.zip › Supplementary Figure SXXX/Supplementary Figure S7.pdf]
